# Insights into the underlying pathogenesis and therapeutic potential of endoplasmic reticulum stress in degenerative musculoskeletal diseases

**DOI:** 10.1186/s40779-023-00485-5

**Published:** 2023-11-09

**Authors:** Ze-Qin Wen, Jun Lin, Wen-Qing Xie, Yun-Han Shan, Ge-Hua Zhen, Yu-Sheng Li

**Affiliations:** 1 Department of Orthopaedics, Xiangya Hospital, Central South University, Changsha, 410008 China; 2 Xiangya School of Medicine, Central South University, Changsha, 410013 China; 3 Department of Orthopaedics, Suzhou Dushu Lake Hospital, Dushu Lake Hospital Affiliated to Soochow University, Medical Center of Soochow University, Suzhou, 215001 China; 4 National Clinical Research Center for Geriatric Disorders, Xiangya Hospital, Central South University, Changsha, 410008 China; 5 Department of General Surgery, Xiangya Hospital, Central South University, Changsha, 410008 China; 6 Department of Orthopaedic Surgery, School of Medicine, Johns Hopkins University, Baltimore, MD 21205 USA

**Keywords:** Endoplasmic reticulum stress, Degenerative musculoskeletal diseases, Pathogenesis, Treatment

## Abstract

Degenerative musculoskeletal diseases are structural and functional failures of the musculoskeletal system, including osteoarthritis, osteoporosis, intervertebral disc degeneration (IVDD), and sarcopenia. As the global population ages, degenerative musculoskeletal diseases are becoming more prevalent. However, the pathogenesis of degenerative musculoskeletal diseases is not fully understood. Previous studies have revealed that endoplasmic reticulum (ER) stress is a stress response that occurs when impairment of the protein folding capacity of the ER leads to the accumulation of misfolded or unfolded proteins in the ER, contributing to degenerative musculoskeletal diseases. By affecting cartilage degeneration, synovitis, meniscal lesion, subchondral bone remodeling of osteoarthritis, bone remodeling and angiogenesis of osteoporosis, nucleus pulposus degeneration, annulus fibrosus rupture, cartilaginous endplate degeneration of IVDD, and sarcopenia, ER stress is involved in the pathogenesis of degenerative musculoskeletal diseases. Preclinical studies have found that regulation of ER stress can delay the progression of multiple degenerative musculoskeletal diseases. These pilot studies provide foundations for further evaluation of the feasibility, efficacy, and safety of ER stress modulators in the treatment of musculoskeletal degenerative diseases in clinical trials. In this review, we have integrated up-to-date research findings of ER stress into the pathogenesis of degenerative musculoskeletal diseases. In a future perspective, we have also discussed possible directions of ER stress in the investigation of degenerative musculoskeletal disease, potential therapeutic strategies for degenerative musculoskeletal diseases using ER stress modulators, as well as underlying challenges and obstacles in bench-to-beside research.

## Background

Degenerative musculoskeletal diseases refer to the structural and functional failures of muscle, bone, cartilage, joint, and surrounding connective tissue, resulting in weakness and motor dysfunction in patients [1]. Osteoarthritis (OA), osteoporosis (OP), intervertebral disc degeneration (IVDD), and sarcopenia (SP) are common musculoskeletal degenerative diseases with similar age-related risk factors [2–5]. OA, the most common degenerative joint disease globally, is characterized by pain and constrained activity, leading to a significant reduction in the quality of life [2, 6]. OP is a dominating public health concern worldwide with distinguishing features of reduced bone mass, microstructural degeneration, decreased bone rigidity, and high risk of fracture [3, 7]. As the major contributor to low back pain and disability, IVDD is generally believed to be caused by the structural damage and low-grade inflammation of intervertebral disc (IVD) elicited by senility [4, 8]. SP is a systemic and deteriorating skeletal muscle disease that clinically manifests an accelerated decrease in muscle mass and strength, further increasing the risk of physical function decline [5]. With the acceleration of the global aging process, degenerative musculoskeletal diseases are becoming increasingly prevalent, which has created additional social and economic burden. Meanwhile, the rapid development of the economy and society promotes the human desire for health and longevity, making aging research unprecedentedly prosperous [9]. Unfortunately, there is still a lack of treatments for delaying or halting the progression of degenerative musculoskeletal diseases in clinical practice. Although a large number of studies have been carried out and a series of tremendous advances have emerged in the past few decades, the detailed pathogenesis of degenerative musculoskeletal disease is still not fully understood, hindering the research and development of effective and safe therapeutic approaches.

Endoplasmic reticulum (ER) is mainly responsible for protein synthesis, folding, and structural maturation, maintaining the activity and function of more than one-third of proteins in eukaryotic cells [10]. ER stress occurs when the aforementioned faculties of the ER are impaired and brings about the accumulation of misfolded or unfolded proteins in the ER [11]. Of note, since the ER is in charge of intracellular calcium (Ca^2+^) storage, perturbations of Ca^2+^ homeostasis can also contribute to ER stress [12]. To protect themselves from ER stress, eukaryotic cells have developed an evolutionarily conserved adaptive mechanism, including the unfolded protein response (UPR) and the ER-associated degradation (ERAD) pathways [13]. Both of these intracellular adaptive responses are triggered by ER stress in response to malfunctions during protein synthesis, folding, and structural maturation, serving as protein quality control programs [14]. Upon mild ER stress, the UPR and ERAD are activated and restore ER homeostasis by removing misfolded and unfolded proteins. However, excessive ER stress beyond the compensatory capacity of UPR and ERAD pathways gives rise to cellular dysfunction and even cell death [14]. Given its decisive role in cellular function and fate, ER stress has increasingly emerged as a significant contributor to the pathogenesis of various diseases, including neurodegenerative disorders, atherosclerosis, diabetes, and cancers [11, 15–17]. In recent years, there has been a growing focus on unraveling the implications of ER stress in degenerative musculoskeletal diseases and their treatment.

Herein, we will compile a comprehensive and thorough summary of updated research findings regarding ER stress in the pathogenesis and progression of degenerative musculoskeletal diseases (including OA, OP, IVDD, and SP) to clarify the underlying mechanisms involved and put forward several prospects for future research in this field. We will also discuss the therapeutic potential of treating degenerative musculoskeletal diseases by regulating ER stress and the remaining challenges in bench-to-beside investigations to provide novel strategies and solid rationale for the future development of curative means for degenerative musculoskeletal diseases.

## ER stress signaling pathways

ER is the largest membrane-bound organelle in eukaryotic cells and is mainly responsible for protein and lipid synthesis, detoxification, and intracellular Ca^2+^ storage [18]. The initial steps of protein maturation that occur in the ER are essential for protein synthesis, and more than one-third of proteins in eukaryotic cells are folded and structurally matured in the ER [10]. Because of its robust protein-processing capacity, the ER bears a significant workload in cells with high secretory demands, making these cells more susceptible to ER stress [16]. Usually, pro-inflammatory cytokines, oxidative stress, hypoxia, nutritional deficiency, and low pH easily induce ER stress [6, 14]. Once ER stress is activated by the aforementioned stimuli, intracellular adaptive responses, including UPR and ERAD, are triggered to eliminate misfolded and unfolded proteins and reestablish ER homeostasis [14]. The UPR alleviates ER stress primarily by inhibiting protein synthesis and increasing chaperone expression to aid in the refolding of misfolded and unfolded proteins, whereas ERAD mainly degrades misfolded and unfolded proteins through the ubiquitin-proteasome pathway.

**Fig. 1:**
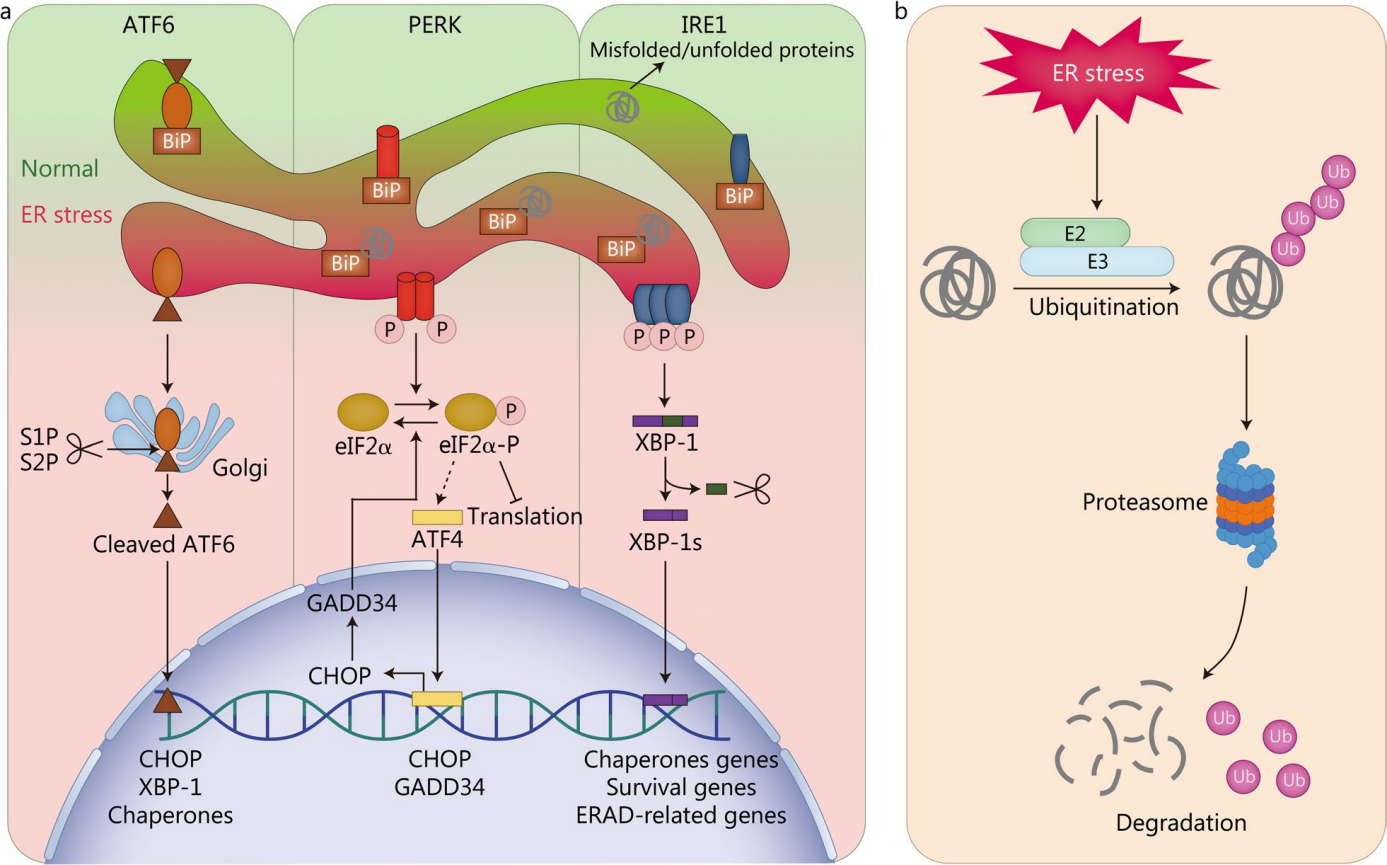
Mechanisms of the UPR (**a**) and ERAD (**b**) pathways. ATF4 activating transcription factor 4, ATF6 activating transcription factor 6, BiP binding immunoglobulin protein, CHOP C/EBP homologous protein, eIF2α α-subunit of eukaryotic translation initiation factor 2, ER endoplasmic reticulum, ERAD ER-associated degradation, GADD34 growth arrest and DNA damage-inducible protein 34, IRE1 inositol-requiring enzyme 1, P phosphorylated, PERK protein kinase R-like ER kinase, S1P site-1 membrane proteases, S2P site-2 membrane proteases, XBP-1 X-binding protein 1, XBP-1s spliced XBP-1, Ub ubiquitin

Generally, three transmembrane proteins located on the ER membrane, including activating transcription factor 6 (ATF6), protein kinase R-like ER kinase (PERK), and inositol-requiring enzyme 1 (IRE1), are responsible for mediating the activation of the UPR induced by ER stress [19] (Fig. 1a). All of these stress sensors of the UPR share a similar structure consisting of an ER luminal domain for sensing stress, a transmembrane domain, and a cytoplasmic domain with enzymatic activity [20]. Glycoprotein 78 kD glucose-regulated protein (GRP78), also known as binding immunoglobulin protein (BiP), is a major ER chaperone [21]. Under physiological conditions, GRP78/BiP binds to the ER luminal domains of ATF6, PERK, and IRE1 to inactivate UPR [19]. Upon the protein-processing function of the ER is impaired, GRP78/BiP dissociates from the stress sensors and interacts with misfolded and unfolded proteins to prevent their aggregation and assist them in refolding accurately, which in turn activates three UPR branches [22]. Furthermore, all the UPR branches can be sensitized by the direct combination of stress sensors to unfolded proteins as well [23].

ATF6 is a transcription factor that is transferred to the Golgi complex and cleaved by site-1 and site-2 membrane proteases upon activation [24]. Cleaved ATF6 exhibits transcriptional activity and facilitates the transcription of genes encoding C/EBP homologous protein (CHOP), X-binding protein 1 (XBP-1), and chaperones in the nucleus [25]. In the second branch of the UPR, translation initiation is blocked by PERK through phosphorylation of the α-subunit of eukaryotic translation initiation factor 2 (eIF2α), which reduces the risk of continued accumulation of misfolded and unfolded proteins to exacerbate ER stress [26]. However, activating transcription factor 4 (ATF4) mRNA is preferentially translated via escaping PERK-mediated translational blockade [27]. Consequently, ATF4 and its downstream target, CHOP, are upregulated whereas other proteins are downregulated [28]. CHOP modulates the expression of several UPR-related genes in response to ER stress by interacting with other transcriptional regulators; it is also a key determinant of cell fate [11, 29]. Growth arrest and DNA damage-inducible protein 34, a substrate-specific regulatory subunit of the holo-phosphatase complex induced by CHOP, can dephosphorylate phosphorylated eIF2α, thereby restoring protein translation and inhibiting ATF4 translation [11]. IRE1 is the most conserved component of the UPR in evolution because it exists in animals, plants, and *Saccharomyces cerevisiae* [30]. In IRE1 branch, misfolded and unfolded proteins are cleared by the upregulation of chaperones and ERAD-related proteins to mitigate ER stress. The cytoplasmic domain of IRE1 possesses serine/threonine kinase and endoribonuclease activities [31]. Upon activation, IRE1 unconventionally splices mRNA encoding XBP-1 through its highly sequence-specific serine/threonine kinase and endoribonuclease activities [32]. Spliced XBP-1 (XBP-1s) encourages the translation of chaperones genes, survival genes, and ERAD-related genes, while unspliced XBP-1 suppresses their translation [23]. IRE1 can also attenuate protein translation and induce cell death by degrading mRNAs localized on the ER membrane through its serine/threonine kinase and endoribonuclease activities, which is called regulated IRE1-dependent decay [33]. Additionally, phosphorylated IRE1 is capable of binding to tumor necrosis factor receptor-associated factor 2 to recruit apoptosis signal-regulating kinase 1, which in turn activates the c-Jun N-terminal kinase (JNK) pathway [34]. The IRE1-apoptosis signal-regulating kinase 1-JNK pathway is activated by excessive ER stress and is closely related to apoptosis [35].

In the ERAD pathway, misfolded and unfolded proteins are transported into the cytoplasm and ubiquitinated by E2 ubiquitin-conjugating enzymes and E3 ubiquitin ligases [36]. Subsequently, the cytoplasmic 26 S proteasome system recognizes and degrades misfolded and unfolded proteins labeled by ubiquitination [36] (Fig. 1b). Apart from the UPR and ERAD, ER stress activates autophagy, which is another protein degradation pathway [37]. During autophagy, damaged macromolecules and organelles are encapsulated in vesicles and transported to lysosomes for degradation and recycling [13]. Excessive ER stress or suppressed UPR, ERAD, and autophagy may contribute to cellular dysfunction or apoptosis.

## ER stress in the pathogenesis of OA

**Fig. 2:**
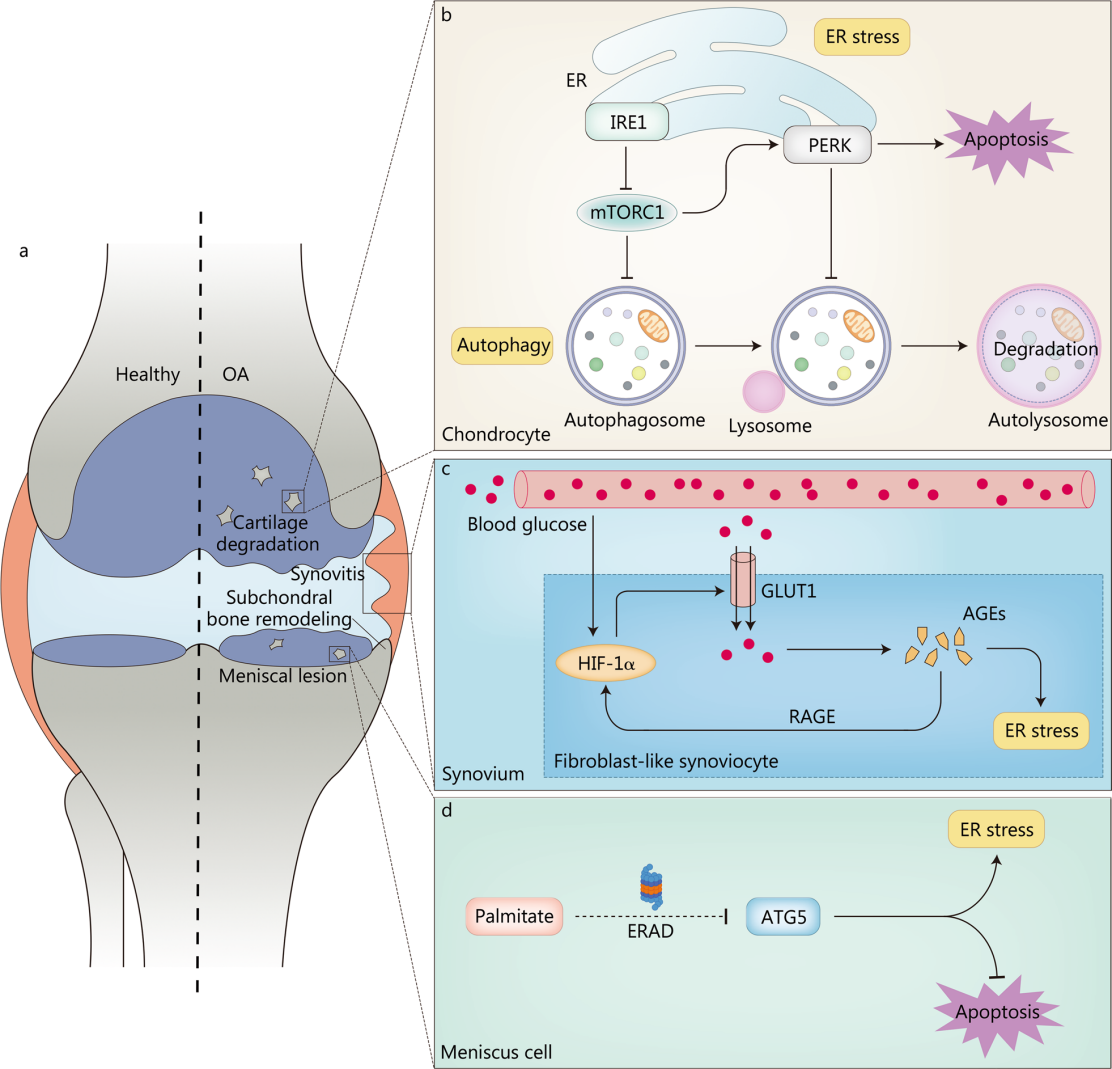
ER stress in the pathogenesis of OA. **a** Pathological changes in OA regulated by ER stress. **b** IRE1-mTORC1-PERK signaling pathway synergistically regulates autophagy and apoptosis in OA chondrocytes. Attenuation of IRE1 signaling activates mTORC1, converting protective autophagy to apoptosis. **c** Mechanisms through which ER stress accelerates OA progression in synovitis. A vicious cycle of HIF-1α-GLUT1-AGEs-HIF-1α may exist in the fibroblast-like synoviocyte of patients with diabetes-related OA, aggravating OA progression. **d** ER stress is involved in meniscal lesion to accelerate OA progression. Palmitate degrades ATG5 through the ERAD pathway to inhibit autophagy and promote meniscus cell apoptosis. AGEs advanced glycation end products, ATG5 autophagy-related 5, ER endoplasmic reticulum, ERAD ER-associated degradation, GLUT1 glucose transporter 1, HIF-1α hypoxia-inducible factor-1α, IRE1 inositol-requiring enzyme 1, mTORC1 mechanistic target of rapamycin complex 1, OA osteoarthritis, PERK protein kinase R-like ER kinase, RAGE receptor for AGEs

As the most prevalent joint disease worldwide, OA is the leading causation of pain and disability [2]. Although previously considered as a degenerative disease of cartilage, increasing evidence has transformed the general understanding of OA into a whole-joint disease covering cartilage degeneration, synovitis, meniscal lesion, subchondral bone remodeling, ligament degeneration, and skeletal muscle degeneration [38] (Fig. 2a). The risk factors for OA are numerous, including aging, obesity, gender, diabetes, and injury, among which aging is the most critical [39]. About 10% of men and 18% of women over 60 years old are affected by OA, and the prevalence of OA increases rapidly with age, resulting in an enormous socioeconomic burden due to increased medical needs and disability-induced early retirement [2, 6]. So far, the clinical treatment for OA is symptomatic and mainly consists of pain management in the early stages and artificial joint replacement in the advanced stages, without disease-modifying drugs currently available for OA [40]. In spite of the rapid development of research on the pathogenesis and treatment of OA, a limited amount of progress is available for clinical use. In recent years, many studies have found an increase in ER stress biomarkers—GRP78, CHOP, ATF6, PERK, and IRE1—in cartilaginous tissues of patients with OA and have reported that this increase is positively correlated with cartilage degeneration [41, 42]. Furthermore, modulation of ER stress using ER stress inhibitors or small-molecule chemical chaperones can protect against OA in vitro and in vivo [43, 44]. These above results enlighten that ER stress is as least partially involved in OA pathogenesis. In the following sections, we will elaborate on the roles of ER stress in the pathogenesis of cartilage degeneration, synovitis, meniscal lesion, and subchondral bone remodeling, respectively.

### ER stress in cartilage degeneration

Cartilage degeneration is the predominant pathological change in OA that causes OA progression [45]. Cartilage is an avascular tissue without sufficient nutrients and supplying cells, making the repair of degenerative cartilage extremely burdensome [46]. Chondrocytes are the only resident cells in cartilage and are primarily responsible for regulating the synthesis-degradation balance of the extracellular matrix (ECM) [47]. Disturbance of this balance caused by chondrocyte dysfunction or death can result in cartilage degeneration. Thus, as a switch that determines the function and fate of chondrocytes, ER stress may play a dominant role in the onset and progression of OA [6]. Insulin-like growth factor-1 (IGF-1) is a growth factor that increases ECM synthesis, including type II collagen, proteoglycans, and other matrix components [48]. ER stress-induced upregulation of tribbles homolog 3 reduced the sensitivity of chondrocytes to IGF-1 by inhibiting protein kinase B (Akt) [49]. In addition, ER stress triggered by palmitate repressed the chondrogenic function of IGF-1 in chondrocytes by activating JNK [50]. Other than inhibiting ECM synthesis, ER stress accelerated ECM degradation in cartilage by upregulating matrix metallopeptidase-13 expression via the p38 mitogen-activated protein kinase (MAPK) pathway [51]. Furthermore, as a biomarker of ER stress, CHOP has been shown to upregulate the expression of sirtuin1 (SIRT1) by directly acting on the promoter region of SIRT1 or indirectly promoting the activation of AMP-activated protein kinase-α, ultimately inhibiting chondrogenic differentiation and ECM production [52].

Moderate ER stress could protect chondrocytes from apoptosis by activating autophagy through the GRP78 pathway, whereas severe ER stress could boost chondrocyte apoptosis, revealing a complicated crosstalk between ER stress, autophagy, and apoptosis [53]. To figure out the mechanisms involved, Yang et al. [54] proposed and demonstrated that the IRE1-mechanistic target of rapamycin complex 1 (mTORC1)-PERK signaling pathway synergistically regulated autophagy and apoptosis in OA temporomandibular joint chondrocytes (Fig. 2b). IRE1-mediated autophagy and PERK-mediated apoptosis were activated simultaneously in response to anomalous mechanical loading. However, IRE1 signaling diminished over time, leading to the activation of mTORC1, which subsequently activated PERK and inhibited autophagosome formation. The sensitized PERK resulted in apoptosis and restrained the fusion of autophagosome and lysosome. Furthermore, decreased Dishevelled, EGL-10 and pleckstrin domain-containing mechanistic target of rapamycin-interacting protein inhibited translocation in renal cancer from chromosome 8 ubiquitination-proteasome degradation in OA chondrocytes, leading to the accumulation of translocation in renal cancer from chromosome 8, excessive ER stress, and OA progression [55]. Ubiquitin-specific peptidase 7, a widely studied deubiquitinase, promoted chondrocyte proliferation and suppressed tumour necrosis factor alpha (TNF-α)-induced apoptosis and inflammation via inhibiting eIF2α-ATF4-CHOP signaling and nuclear factor-kappa B (NF-κB)/p65 signaling, thereby preventing the occurrence of OA [56, 57].

Among the numerous risk factors for OA, aging is the most significant [58]. And the prevalence of OA increases sharply with age [59]. Many hypotheses have revealed the processes by which aging leads to OA, and ER stress may be one of them, serving as a bridge linking aging and OA. Aging is closely associated with decreased chaperones, chaperone dysfunction, perturbations of critical regulatory molecules in ER stress, and increased senescence-related biomarkers, all of which can elicit excessive ER stress [60–64]. Tan et al. [65] compared knee cartilage obtained from young (6 to 11 years old) and aged cynomolgus monkeys (20 to 34 years old), and found that the expression of chaperones was downregulated in aged cynomolgus monkeys, while ER stress and apoptosis markers were upregulated and could be partially restored by PERK inhibitor. Senescence-related dysfunction of chaperones also contributed to ER stress, for example, BiP ATPase activity and protein disulfide isomerase enzyme function were significantly reduced in hepatocytes of senescent mice [66]. However, whether a similar mechanism exists in chondrocytes remains to be verified. Furthermore, *PERK* mRNA expression was statistically decreased in the hippocampus of senescent rats and increased the risk of ER stress [61]. Senescence-associated β-galactosidase, the most widely used biomarker of senescence, was increased in OA chondrocytes and decreased after the inhibition of ER stress [62, 67]. Advanced glycation end products (AGEs), a group of compounds synthesized by nonenzymatic glucose-protein condensation (also known as the Maillard reaction), are inevitable products of senescence and accumulate in various cells [63, 68]. Yamabe et al. [64] unveiled that the accumulation of AGEs in chondrocytes induced ER stress and apoptosis by modifying UPR-related proteins, thereby hastening OA progression. Rasheed et al. [69] further demonstrated that AGEs mediated ER stress in human chondrocytes via eIF2α, p38 MAPK, and NF-κB pathways. Nevertheless, the associations between ER stress and other senescence-related biomarkers or senescence-associated secretory phenotype (a toxic microenvironment in which cells stop dividing and begin to secrete chemokines, cytokines, and extracellular matrix proteins, resulting in DNA damage) in chondrocytes still wait for further exploration [70].

As mentioned earlier, obesity is another pathogenic factor of OA. The mechanisms of obesity causing OA may be the accumulation of free fatty acids in non-adipose tissues, which is called lipotoxicity, rather than mechanical-associated pathogenesis [71, 72]. Tan et al. [73] observed that high-fat diet facilitated chondrocyte apoptosis and OA progression by mediating ER stress. Interestingly, the type of dietary fat affects the onset of OA caused by obesity. Palmitate (a kind of saturated fatty acid) instead of oleate (a kind of monounsaturated fatty acid) promoted chondrocyte apoptosis by inducing ER stress both in vivo and in vitro [74, 75]. Glucagon-like peptide-1 (GLP-1) is an incretin hormone secreted by intestinal L cells that regulates energy metabolism homeostasis by combining with GLP-1 receptor (GLP-1R) [76]. Chen et al. [77] revealed that the activation of GLP-1R with liraglutide could inhibit ER stress and apoptosis by activating the phosphatidylinositol-4,5-bisphosphate 3-kinase/Akt pathway in vitro and mitigate cartilage degeneration in vivo.

Notably, gender is also reported to be one of the risk factors for OA [78]. Incidence of OA in women increases significantly around the age of 50 and knee OA is more severe in women after menopause [79, 80]. Dreier et al. [81] found that estrogen at physiological concentrations could inhibit ER stress and ER stress-induced apoptosis in ER protein 57 (ERp57)-knockout C28/I2 chondrocytes. This study preliminarily illuminated that estrogen could affect OA progression by regulating ER stress, however, the concrete mechanisms through which estrogen decreases ER stress and whether gender accelerates OA progression by regulating ER stress in other ways remains undetermined.

### ER stress in synovitis

The synovium consists of two layers: the lining layer is composed of numerous fibroblast-like synoviocytes (FLSs) and synovial macrophages, and the sub-lining layer contains mainly connective tissue and few cellular components [82]. Contrary to cartilage, synovium is rich in vascular, neural, and cellular components, responsible for secreting synovial fluid and maintaining joint homeostasis [83]. The latest view suggests that synovitis may be the initial pathological change preceding cartilage degeneration and is mainly responsible for OA pain [84]. Because the synovium participates more in systemic circulation than cartilage without blood vessels, and is more sensitive to serum modulators, it may be the pathological tissue and curative target of diabetes-related OA [85]. In rat FLSs, high glucose stimulation upregulated the expression of glucose transporter 1 (GLUT1) and the accumulation of AGEs through hypoxia-inducible factor 1α (HIF-1α), thus forwarding ER stress and the release of pro-inflammatory mediators [86]. Besides, the combination of AGEs and the receptor for AGEs can activate HIF-1α signaling in various tissues, hence there may be HIF-1α-GLUT1-AGEs-HIF-1α loop and lead to diabetes-related OA [86, 87] (Fig. 2c). The NOD-like receptor family pyrin domain containing 3 (NLRP3) inflammasome consists of NLRP3, adaptor apoptosis-associated speck-like protein, and procaspase-1, serving as a key factor in initiating the inflammatory cascade of amplification [88]. The upregulation of thioredoxin-interacting protein (TXNIP) elicited by the PERK-CHOP pathway activated the NLRP3 inflammasome [89]. In OA rat models and lipopolysaccharide-treated FLSs, Liu et al. [90] unveiled that the ER stress/TXNIP/NLRP3 signaling pathway could mediate synovitis and the release of pro-inflammatory mediators including interleukin (IL)-1β and IL-18. Moreover, the GRP78-NF-κB pathway is involved in IL-1β-induced pro-inflammatory mediator release from FLSs and synovial macrophage polarization, which are pivotal factors in the pathogenesis of synovitis [91].

### ER stress in meniscal lesion

The meniscus is mainly composed of water (72%) and organic matter (28%), including meniscus cells and ECM [92]. As one of the essential components of joints, meniscus plays a significant role in alleviating OA progression by absorbing shock and stabilizing joints, and is exposed to the biomechanical and biochemical microenvironment similar to cartilage [42, 92]. However, OA has long been considered a degenerative disease of cartilage, rather than a degenerative disease of meniscus [40]. Recently, meniscus has been proposed to be more susceptible to the catabolic effect of adipokines than cartilage, indicating that meniscus may be the major tissue affected in obesity-related OA [93]. However, palmitate, rather than oleate, has been shown to induce ER stress and prompt apoptosis in meniscus cells [42]. Furthermore, palmitate could restrain autophagy and stimulate apoptosis of meniscus cells by degrading autophagy-related 5 (ATG5) through ERAD pathway [94] (Fig. 2d).

### ER stress in subchondral bone remodeling

Subchondral bone usually refers to the bony component located at the distal end of calcified cartilage, which consists of subchondral bone plate and trabecular bone [95, 96]. Remodeling of the subchondral bone microstructure is closely related to cartilage degeneration during OA progression [97]. Subchondral bone and cartilage contribute to subchondral bone remodeling and cartilage degeneration through biomechanical coupling and interactions, forming a vicious cycle accelerating OA progression [98]. Targeted inhibition of subchondral bone remodeling relieves OA symptoms and protects cartilage from degradation [99]. Osteoblasts, osteoclasts, osteocytes, and bone marrow mesenchymal stem cells (BMSCs) all participate in subchondral bone remodeling [100]. Among the four cell types, the coupling of osteoblast-mediated bone formation and osteoclast-mediated bone resorption mainly regulate subchondral bone remodeling [101]. Subchondral bone remodeling in OA is usually accompanied by containment of osteoblast mineralization, which can be partially restored by inhibiting ER stress [102]. PERK mediates osteoclast differentiation and bone resorption, while inhibiting PERK curtails osteoclastogenesis and mitigates bone loss in ovariectomized mouse models [103]. Osteocytes are the terminally differentiated osteoblasts embedded in the bone matrix, which affect osteoclastogenesis by regulating the expression of receptor activator of NF-κB ligand (RANKL) in response to mechanical stimuli [100]. Inhibition of ER stress alleviates cell death and diminishes intracellular reactive oxygen species (ROS) levels in osteocytes induced by oxygen-glucose deprivation [104]. Because osteocytes are as old as the embedded bone packet, osteocytes may be more susceptible to excessive ER stress compared to the short-lived osteoblasts and osteoclasts [105]. However, no experiments have been performed to confirm whether cellular lifespan affects ER stress. BMSCs are a heterogeneous population of cells with self-renewal capacity and multidirectional differentiation potential [106]. ER stress and impaired autophagy elicited inflammation-mediated bone loss via the activation of Rho-associated protein kinase 1 in BMSCs [107].

### ER stress in ligament degeneration

Ligament connects two bones or fibrous cartilages to bone and functions by balancing abnormal mechanical forces within the joint [108]. Furthermore, because ligament comprises a mass of ECM, poor blood supply, and low cellular contents, it is prone to degeneration and tears (especially aging ligaments with lower cellular components) [108]. Impaired ligament can destabilize the joint and accelerate OA progression. Ligament containing free nerve endings are involved in OA pain along with other innervated tissues including synovium [109]. Li et al. [110] observed that dexamethasone led to calcification and degeneration of anterior cruciate ligament cells through ER stress, increasing the risk of injury. In addition, Shi et al. [111] found that ER stress might facilitate mechanical stress-induced ossification of the posterior longitudinal ligament through the MAPK signaling pathway. Unfortunately, research on the roles of ER stress in ligament degeneration of OA is lacking.

### ER stress in skeletal muscle degeneration

Skeletal muscle structure and function are related to the onset and progression of OA [112]. Currently, relevant study has concentrated on the relationship between muscle strength and OA progression [112]. In addition to skeletal muscle function, skeletal muscle constitution, biochemical and molecular interactions are considered to be the underlying mechanisms [112]. Kim et al. [113] reported that valdecoxib, a non-steroidal anti-inflammatory drug, could relieve lipid-induced skeletal muscle insulin resistance by inhibiting inflammation and ER stress. However, there have been no studies on ER stress in the skeletal muscle degeneration of OA.

### ER stress in OA neuropathic pain

Pain is the most significant symptom of OA and a major determinant of clinical decision-making and patient counseling [114]. Poor pain management is also the main cause of disability and artificial joint replacement in OA patients [115]. OA pain includes nociceptive, inflammatory, and neuropathic pain (referring to pain caused by diseases of the central and peripheral nervous systems). ER stress in the peripheral nervous system has been identified as an important driver of neuropathic pain in diabetes [116]. Mao et al. [117] demonstrated that ER stress contributed to bone cancer pain in a mouse model and inhibition of ER stress in spinal neurons relieved bone cancer pain by modulating neuroinflammation. Nevertheless, there are few studies on the role of ER stress in OA neuropathic pain.

## ER stress in the pathogenesis of OP

OP is a systemic skeletal disorder characterized by reduced bone mass, microstructural degeneration, decreased bone rigidity, and high risk of fracture [7]. Risk factors of OP include aging, menopause, iatrogenic factors (excessive use of glucocorticoids is the most common), behavior, nutrition, and genetics [3]. Due to the aging of the world population, OP is estimated to affect 200 million people globally, especially those over 60 years of age [118]. Osteoporotic fracture is the most serious clinical consequence of OP, with hip fracture being the most dangerous [3]. Statistically, one in five people die in the first year after a hip fracture, and less than a third fully return to normal [3]. In the United States, more than 1.5 million people are diagnosed with osteoporotic fractures each year, at a cost of about $17 billion and projected to reach $50 billion annually by 2040 [3]. The magnitude of socioeconomic burden and health risks urge researchers to explore the pathogenesis of OP and develop effective curative tactics. Due to the relatively high demand of osteoblasts to secrete bone matrix, ER stress may determine the cell function and fate of osteoblasts, and play a decisive role in the progression of OP. In the following, we will summarize the latest findings that ER stress not only affect osteoblasts, but also osteoclasts, osteocytes, BMSCs, and vascular endothelial cells to mediate OP pathogenesis.

### ER stress in bone remodeling

**Fig. 3:**
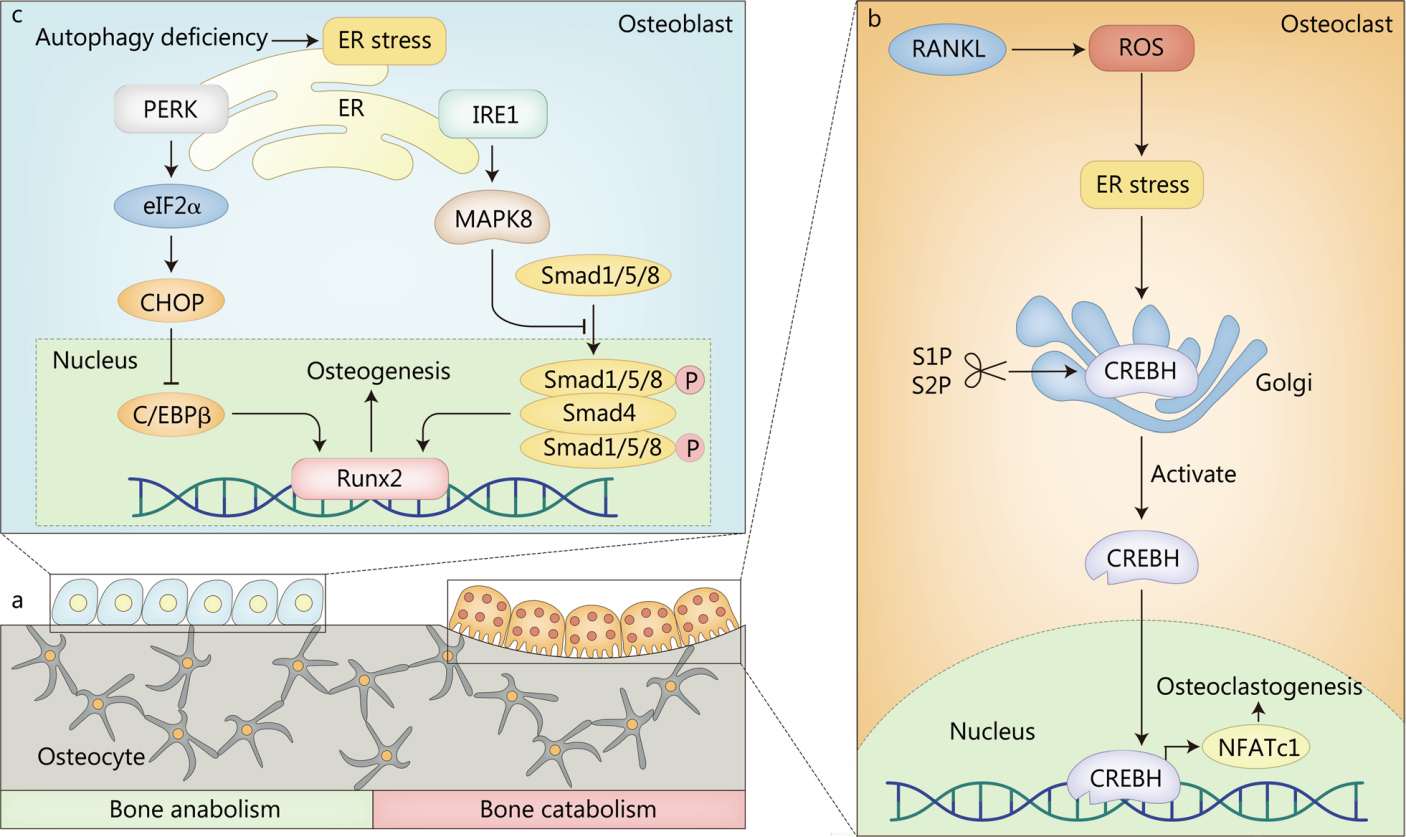
ER stress in the pathogenesis of OP. **a** Bone remodeling is mainly regulated by the coupling of osteoblast-mediated bone formation and osteoclast-mediated bone resorption, and osteocytes have also been reported to participate in bone remodeling in OP. **b** RANKL activates CREBH through ROS/ER stress signaling pathway to promote the transcription of NFATc1, ultimately leading to increased osteoclastogenesis. **c** Autophagy deficiency induced by conditional *Atg7* deletion inhibits mineralization and promotes ER stress and apoptosis in osteoblasts. CHOP C/EBP homologous protein, CREBH cAMP response element-binding protein H, C/EBPβ CCAAT/enhancer binding protein β, eIF2α α-subunit of eukaryotic translation initiation factor 2, ER endoplasmic reticulum, IRE1 inositol-requiring enzyme 1, MAPK8 mitogen-activated protein kinase 8, NFATc1 nuclear factor of activated T cells cytoplasmic 1, PERK protein kinase R-like ER kinase, RANKL receptor activator of NF-κB ligand, ROS reactive oxygen species, Runx2 Runt-related transcription factor 2, S1P site-1 membrane proteases, S2P site-2 membrane proteases, Smad small mother against decapentaplegic

Bone remodeling constitutes a dynamic equilibrium between bone formation and resorption, serving as the primary mechanism for bone renewal and adaptation to changes in load-bearing demands. This intricate process hinges predominantly on the synergy of osteoblast-mediated bone formation and osteoclast-mediated bone resorption, accompanied by the regulatory influence of osteocytes [119] (Fig. 3a). Both decreased bone formation and increased bone resorption during bone remodeling contribute to OP. Therefore, ER stress-induced cellular dysfunction and aberrant apoptosis of osteoblasts, osteoclasts, and osteocytes might underlie the development of OP. Indeed, ER stress has been shown to participate in OP by inducing osteoblast apoptosis and facilitating osteoclast differentiation, migration, and adhesion, as observed both in vivo and in vitro [120]. Moreover, considering the capacity of BMSCs and bone marrow hematopoietic cells to differentiate into osteoblasts and osteoclasts, it is plausible that ER stress could play a role in the initiation and progression of OP by modulating their differentiation processes.

The signal transducer and activator of transcription 3 is a transcription factor boosting cell survival and differentiation [121]. Signal transducer and activator of transcription 3 were reported to upregulate the expression of miR-205, which could directly target the 3’-untranslated region of the *CHOP* gene to downregulate its expression and in turn, inhibited the apoptosis of osteoblasts [122]. In addition, GRP78 was increased in osteoblasts and adipocytes of aged and ovariectomy-induced OP mice, and GRP78 negatively regulated osteoblast differentiation and facilitated adipogenic differentiation in C3H10T1/2 cells [123]. Glutathione peroxidase 7 is a highly conserved member of the glutathione peroxidase family that functions as an antioxidant together with superoxide dismutase [124]. Glutathione peroxidase 7 deficiency might suppress the mechanistic target of rapamycin pathway by upregulating ER stress, thereby reducing the osteogenic potential of BMSCs [125].

RANKL is essential for osteoclast differentiation, survival, and activation [126]. RANKL binds to the receptor activator of NF-κB located on the membrane of osteoclast precursors, leading to the activation of c-Fos, NF-κB, and nuclear factor of activated T cells cytoplasmic 1 (NFATc1) by recruiting signaling adaptor molecules tumor necrosis factor receptor-associated factor [127]. NFATc1 in turn upregulates the expression of various osteoclast-specific genes and is indispensable for osteoclast differentiation [128]. Lee et al. [129] testified a crucial role of excessive ER stress in RANKL-induced NFATc1 activation and osteoclast differentiation mediated by IL-1β. cAMP response element-binding protein H is a transcription factor activated by UPR-dependent proteolytic cleavage and then translocated to the nucleus to regulate the transcription of target genes [130]. RANKL activated cAMP response element-binding protein H through the ROS/ER stress signaling pathway to prompt the transcription of NFATc1, ultimately leading to increased osteoclastogenesis [126] (Fig. 3b).

Similar to chondrocytes, a tangled crosstalk of ER stress, autophagy, and apoptosis exists in osteoblasts and osteoclasts as well. Yang et al. [131] reported that ER stress attenuated oxidative damage and apoptosis in osteoblasts by enhancing autophagy. Autophagy-related 7 (ATG7) is an essential autophagy effector enzyme that acts in coordination with other ATG proteins to regulate autophagy, apoptosis, and cell cycle [132]. Li et al. [133] found that autophagy deficiency induced by conditional *Atg7* deletion inhibited mineralization, and promoted ER stress and apoptosis in osteoblasts. Bone morphogenetic protein signaling, a key regulator of embryonic bone development and postnatal bone homeostasis, binds to its receptor and functions through both small mother against decapentaplegic (Smad)-dependent and Smad-independent signaling pathways [134]. In the Smad-dependent pathway, two phosphorylated R-Smads (Smad1/5/8) bind to a co-Smad (Smad4) and co-translocate to the nucleus, where they recruit Runt-related transcription factor 2 (Runx2) to regulate osteoblast-specific gene expression. PERK activation induced by autophagy deficiency upregulated the expression of CHOP by phosphorylating of eIF2α, and the increased CHOP formed a heterodimer with CCAAT/enhancer binding protein β (C/EBPβ) to inhibit the Runx2-binding activity of C/EBPβ, leading to the downregulation of osteogenic gene expression [133]. Moreover, the activation of IRE1 triggered by autophagy deficiency inhibited the bone morphogenetic protein-Smad1/5/8 pathway via MAPK8, thereby constricting Runx2-mediated osteoblast-specific gene expression (Fig. 3c). Furthermore, site-1 membrane proteases (S1P) depletion in bone marrow monocytes inhibited ATF6 and sterol response element binding protein 2 (SREBP2) maturation, which subsequently blocked CHOP/SREBP2 complex-induced autophagy, thereby suppressing osteoclast differentiation [135].

OP can be categorized into primary (including the senile and postmenopausal OP) and secondary OP (including glucocorticoids-induced OP), with primary OP being more common [136]. Senile OP, which occurs in the elderly over 70 years old, has now become a focus of global health care concern. The pathogenesis of senile OP has not been fully elucidated, and cellular senescence is a possible mechanism. Suzuki et al. [137] observed that AGEs accumulated in osteoblasts with aging could elicit apoptosis through ER stress. However, the association between ER stress and other senescence-associated biomarkers (such as senescence-associated β-galactosidase) or senescence-associated secretory phenotype, remains unclear. Postmenopausal OP is caused by estrogen deficiency in postmenopausal women. 17β-estradiol, a major estrogen, was reported to protect osteoblasts from ER stress-induced apoptosis by promoting Ras-ERK1/2 signaling pathway dependent transcription factor II-I activity to upregulate the expression of GRP78, which could inhibit apoptosis by binding to procaspases-7 and procaspases-12 [138]. This contradicts the typical role of GRP78 as a pro-apoptotic molecule for reasons that are still not known. Glucocorticoids are widely used in inflammatory and autoimmune diseases, and long-term administration of large amounts of glucocorticoids may lead to glucocorticoids-induced OP [139]. Glucocorticoids accelerated the apoptosis of osteoblasts and osteocytes partly through ER stress, and the inhibition of eIF2α dephosphorylation protected osteoblasts and osteocytes from apoptosis induced by glucocorticoids in vitro and in vivo [140]. Further study has shown that glucocorticoids induce ER stress-mediated apoptosis by increasing CHOP expression in osteoblasts [141].

In addition, due to the global epidemic of diabetes, the morbidity of diabetic OP caused by chronic hyperglycemia, AGEs, and oxidative stress has gradually increased [142]. ER stress was implicated in the progression of diabetic OP in vivo and in vitro by increasing CHOP expression in osteoblasts [143]. Obesity has recently been found to be strongly associated with the onset of OP, which may be an addition risk factor for OP [144]. In obese patients, serum free fatty acids are often increased due to lipid accumulation and reduced utilization of fatty acids (FAs), leading to lipotoxicity in BMSCs and osteoblasts [145]. Palmitate was proven to induce the apoptosis of BMSCs and osteoblasts through ER stress, which was alleviated by oleate [146]. Homocysteine, a sulfur-containing amino acid, is now considered a risk factor for OP and is commonly found in hyperhomocysteinemia, an inherited disorder of amino acid metabolism [147]. Park et al. [147] observed that homocysteine induced apoptosis through ER stress in osteoblasts. Besides, exercise is known to increase bone rigidity by improving bone mineral content and structure, and the mechanism is thought to be at least through affecting osteogenic and adipogenic differentiation balance of BMSCs [148]. Styner et al. [149] reported that mechanical stimulation upregulated BiP and downregulated CHOP expression to alleviate ER stress and inhibit adipogenesis by inhibiting C/EBPβ expression in BMSCs. However, C/EBPβ overexpression did not restore the suppression of adipogenesis by mechanical stimulation, suggesting that mechanical sensitivity of C/EBPβ is not the primary site of adipogenesis regulation in BMSCs. In addition, ER stress reduced the response of osteocytes to mechanical stimulation and then contributed to low bone mass [150].

Bone marrow hematopoietic cells, including macrophages and neutrophils, exert an indirect influence on bone remodeling [151, 152]. Exhibiting remarkable heterogeneity and plasticity, macrophages shift from non-activated M0 macrophages to pro-inflammatory M1 phenotype when triggered by lipopolysaccharide or interferon-γ, while they can alternatively assume an anti-inflammatory M2 phenotype under the influence of IL-4, IL-13, or IL-10 [153–155]. The co-culture of M1 and M2 macrophages enhanced the osteogenic capacity of MC3T3 cells, as demonstrated by increased alkaline phosphatase activity and matrix mineralization [156]. After IL-4 treatment, which induced the repolarization of M1 macrophages into the M2 subset, the osteogenic capacity of M1-MC3T3 co-cultures was further elevated [156]. Interestingly, the direct effect of IL-4 on MC3T3 cells did not result in an increase in osteogenic capacity, implying that the increased osteogenic capacity could potentially be ascribed to the secretion of osteoblastic cytokines by M2 macrophages [157]. Within tumor cells, ER stress promoted the repolarization of macrophages into the M2 phenotype by modulating the secretion of extracellular vesicles [158, 159]. The SIRT1/ER stress signaling pathway was also recognized to participate in the modulation of macrophage polarization [160]. However, further investigations are required to establish whether ER stress can impact the progression of OP by regulating macrophage polarization. Neutrophils, a vital cell type within the innate immune system, arise from hematopoietic stem cells and progenitor cells in the bone marrow [161]. Within the context of rheumatoid arthritis, activated neutrophils contributed to osteoclast-mediated bone resorption by promoting the expression of RANKL in inflamed joints [162, 163]. Moreover, neutrophils expressing RANKL were increased in male patients with chronic obstructive pulmonary disease and were associated with low bone density, suggesting their potential role in bone resorption [164]. One of the three branches of ER stress, IRE1α, plays a crucial role in the heightened activity of neutrophils in lupus [165]. Nonetheless, whether ER stress contributes to OP by influencing the function and fate of neutrophils remains to be conclusively determined.

### ER stress in angiogenesis

The onset of OP is associated with a decrease in bone marrow microvessels, and the encouragement of angiogenesis exhibited therapeutic effect on OP [166]. Therefore, ER stress may accelerate OP progression by mediating dysfunction or apoptosis of vascular endothelial cells and BMSCs. Naringin inhibited the apoptosis of vascular endothelial cells by blocking ER stress, which might delay OP progression [167]. Bisphosphonates are the most common anti-resorptive drugs used to treat OP. Alendronate is a nitrogen-containing bisphosphonate that exerts an anti-resorptive effect by binding to and inhibiting the activity of farnesyl diphosphate synthase [168]. Recent study has shown that preosteoclasts hardly resorb bone matrix and even facilitate angiogenesis by secreting platelet-derived growth factor-BB [169]. Ding et al. [170] proposed that the lack of selectivity of alendronate in inhibiting preosteoclasts and mature osteoclasts could lead to peroxisome dysfunction in preosteoclasts, further inducing ER stress and apoptosis. Since decreased angiogenesis increases the risk of OP, how to retain proangiogenic preosteoclasts while depleting mature osteoclasts is a problem worth investigating, and targeted drug delivery systems may be a suitable solution.

## ER stress in the pathogenesis of IVDD

**Fig. 4:**
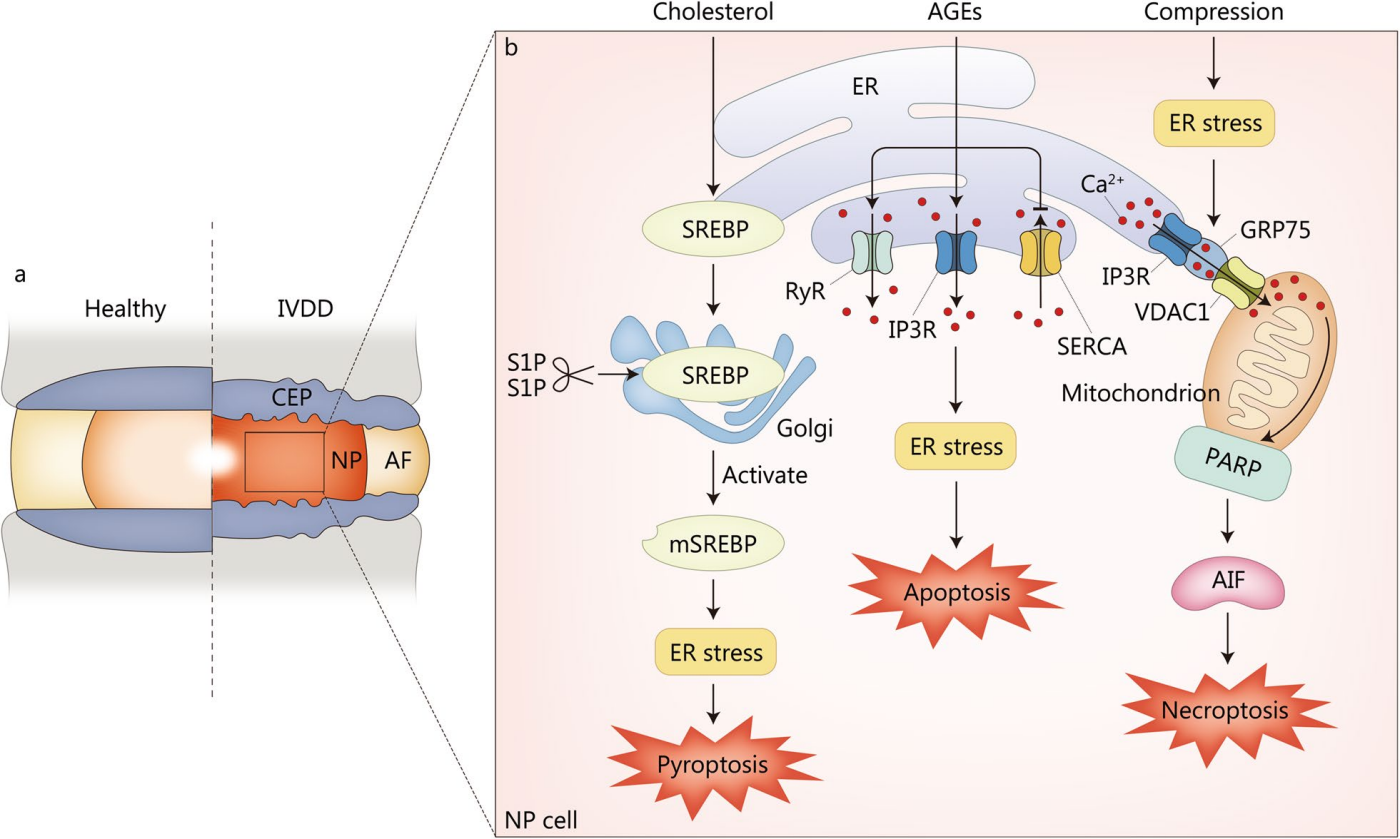
ER stress in the pathogenesis of IVDD. **a** Pathological changes of IVDD involved by ER stress. **b** The mechanisms by which ER stress promotes IVDD progression through aggravating NP degeneration. IP3R is located on ER, VDAC1 is a mitochondrial outer membrane protein, and GRP75 is a connexin, which can link IP3R to VDAC1 to form a channel for Ca^2+^ translocation. AF annulus fibrosus, AGEs advanced glycation end products, AIF apoptosis-inducing factor, CEP cartilaginous endplate, ER endoplasmic reticulum, GRP75 glucose-regulated protein 75, IP3R inositol 1,4,5-trisphosphate receptor, IVDD intervertebral disc degeneration, mSREBP mature form of SREBP1, NP nucleus pulposus, PARP poly(ADP-ribose) polymerase, RyR ryanodine receptor, SERCA sarco/endoplasmic reticulum Ca^2+^-ATPase, SREBP sterol response element binding protein, S1P site-1 membrane proteases, S2P site-2 membrane proteases, VDAC1 voltage-dependent anion-selective channel 1

The IVD is the largest avascular fibrocartilaginous organ that acts to absorb shock and provide flexibility to the spine, consisting of the gel-like nucleus pulposus (NP) in the center, the annulus fibrosus (AF) around it, and the cartilaginous endplate (CEP) that anchors the IVD to the corpus vertebrae [171]. IVDD is characterized by the degeneration of local physiological structures, including decreased hydration of the NP, destruction of the AF, and calcification of the CEP, which is a major cause of low back pain [172] (Fig. 4a). Approximately 700 million individuals worldwide suffer from low back pain, which reduces the quality of life of IVDD patients and may eventually lead to disability [173]. At the same time, low back pain caused by IVDD is also the most common reason for patients visits [174]. Increased absence from work, decreased productivity and massive consumption of medical resources caused by low back pain place huge burdens on society [173]. IVDD has many known risk factors, including abnormal mechanical loading, obesity, aging, genetics, and poor diet [8]. ECM in NP mainly consists of proteoglycan and type II collagen, which is highly hydrated and contributes to resisting axial mechanical load; while in AF, ECM is mainly composed of type I collagen, which is helpful to withstand lateral mechanical stimulation [175, 176]. The balance between ECM anabolism and catabolism plays a pivotal role in maintaining IVD homeostasis [172]. Hence, the disturbance of ECM anabolism and catabolism caused by ER stress may lead to the biomechanical imbalance of IVD and accelerate the progression of IVDD. Furthermore, the expression of ER stress biomarkers, such as GRP78 and CHOP, was increased in NP from IVDD patients [177]. The expression of GRP78 and CHOP was positively correlated with the Pfirrmann grades of IVDD [178]. Targeted inhibition of IRE1 restrained the degeneration of NP cells and postponed the progression of IVDD in a puncture rat model [179]. Notably, a recent single-cell RNA sequencing analysis of NP cells from patients with IVDD showed that late degenerative NP cells were predominantly composed of cell types associated with ER stress, inflammatory response, and fibrocartilaginous activity, which might be major contributors to the late progression of IVDD [180]. These findings all suggest that ER stress is involved in the pathogenesis of IVDD.

### ER stress in NP degeneration

NP is the most hydrated part of the IVD and develops from the embryonic notochord [181]. ECM secreted by NP cells, including type I collagen, type II collagen, and proteoglycan, enables IVD to absorb mechanical loading and maintain physiological function [182]. Anomalous mechanical loading beyond IVD affordability is a critical pathogenic factor in the pathogenesis and progression of IVDD [175]. Excessive mechanical loading has been reported to facilitate pro-inflammatory mediator production and apoptosis of NP cells, leading to NP degeneration and IVDD [183, 184]. Xiang et al. [185] observed a significant increase in ER stress markers (GRP78 and CHOP) over time in high-pressure gas-treated NP cells. Moreover, ER stress induced by compression promoted NP cell apoptosis [186]. Interestingly, necroptosis (also called programmed necrosis), a programmed cell death triggered by death receptors independent of caspase, was also caused by compression-induced ER stress in NP cells [186, 187]. Lin et al. [188] further unveiled compression-enhanced ER stress and ER stress-induced Ca^2+^ influx from ER to mitochondria via inositol 1,4,5-trisphosphate receptor-glucose-regulated protein 75-voltage-dependent anion-selective channel 1 complex. Consequently, increased ER-mitochondria Ca^2+^ crosstalk elicited necroptosis of rat NP cells through the poly(ADP-ribose) polymerase/apoptosis-inducing factor pathway (Fig. 4b).

Crosstalk between ER stress, autophagy, and apoptosis is another potential pathogenesis of IVDD. Hydrogen peroxide promoted NP cell apoptosis by stimulating ER stress and ER stress-dependent autophagy via the IRE1/JNK pathway [189]. PERK/eIF2α pathway promoted NP cell survival in response to TNF-α stimulation by activating autophagy [190]. Moderate ER stress and low levels of ROS induced by short-term glucose deprivation protected NP cells from apoptosis through autophagy, whereas excessive ER stress and ROS production caused by late glucose deprivation contribute to the apoptosis of NP cells [191]. These abovementioned findings are suggestive of a double-edged role of ER stress and autophagy in regulating cell survival. Apart from autophagy, another particular type of selective autophagy, ER-phagy (also called reticulophagy), has attracted our attention [192]. Glucose deprivation induced senescence and apoptosis of human NP cells and resulted in IVDD through ER stress [193]. At the same time, enhanced ER stress could induce reticulophagy regulator 1-mediated ER-phagy in response to glucose deprivation, which in turn prevented glucose deprivation-induced cell senescence and apoptosis by inhibiting ER stress in human NP cells [193].

IVDD is associated with increased expression of pro-inflammatory cytokines that promote ECM degradation, chemokine secretion, and alterations in cell phenotype, resulting in imbalance between ECM anabolism and catabolism [4]. Krupkova et al. [194] reported that ER stress mediated IL-6 release via p38 MAPK and CHOP in primary IVD cells. In turn, pro-inflammatory mediators (IL-1β and TNF-α) significantly upregulated IRE1 and PERK expression, but not ATF6, thereby amplifying the inflammatory cascade [195]. TNF-α increased CHOP expression through the JNK/ERK/MAPK and NF-κB signaling pathways, and then promoting NP cell apoptosis [196]. However, in contrast, Chen et al. [197] found that PERK/eIF2α and IRE1/XBP1 enhanced the survival and proliferation of rat NP cells following TNF-α stimulation. This also indicates that ER stress possesses two opposite pro-apoptotic and pro-survival effects.

Hypercholesterolemia is becoming a global public health problem owing to a high-fat diet and an unhealthy lifestyle [198]. Sprague-Dawley rats on a high-cholesterol diet showed more severe IVDD than rats on a normal diet [199]. Statins, a class of drugs recommended by professional guidelines and widely used worldwide to lower blood cholesterol, were found to alleviate IVDD in vivo and in vitro [199–201]. Pyroptosis, unlike apoptosis, is a specific programmed cell death mediated by gasdermin [202]. Yan et al. [199] reported that cholesterol accumulation induced pyroptosis of NP cells via the mature form of SREBP1 (mSREBP1)-mediated ER stress (Fig. 4b). The results of these studies extend our understanding of IVDD pathogenesis and provide a novel therapeutic approach for the IVDD from the perspective of improving systemic metabolic disorders.

Aging is a major risk factor for IVDD, but the pathogenesis leading to IVDD during aging remains uncertain [203]. AGEs accumulate in IVD with age, especially in diabetic patients, leading to increased ECM degradation and cellular apoptosis, which is considered to be one of the mechanisms of aging-induced IVDD [204, 205]. Liao et al. [178] demonstrated that AGEs induced ER stress to promote NP cell apoptosis. Luo et al. [206] further reported that AGEs accumulation resulted in excessive ER stress and the subsequent NP cell apoptosis by increasing the expression of ER-resident Ca^2+^-release channels inositol 1,4,5-triphosphate receptor and ryanodine receptor, and decreasing the expression of ER Ca^2+^-reuptake pumps sarco/ER Ca^2+^-ATPase (Fig. 4b).

The nutrients and oxygen of IVD are mainly derived from the capillary network of CEP [207]. Due to diffusion, the oxygen tension gradually decays from the surrounding AF to the central NP, forming an anoxic microenvironment. As a result, glycolysis becomes the main energy supply mode for NP cells, through which large amounts of lactate are produced and result in a low pH microenvironment [208]. Zhu et al. [209] found that ER stress inhibited acidic microenvironment-induced senescence of NP cells by activating autophagy. Whereas Xie et al. [210] proclaimed that acidic microenvironment promoted apoptosis of NP cells through activation of acid-sensing ion channel 1a and acid-sensing ion channel 1a-mediated ER stress. The different effects of ER stress may be caused by different stimuli to NP cells.

### ER stress in AF rupture

AF is the peripheral structure of IVD with a zonal distribution of phenotypically distinct cells [211]. The ECM produced by the outer AF cells contained abundant type I collagen and a small amount of proteoglycan, while the ECM produced by the inner AF was mainly composed of type II collagen and proteoglycan. The structural integrity of AF is critical for limiting NP movement and maintaining physiological IVD pressure under aberrant mechanical loading, and thus plays a vital role in the biomechanical properties of IVD and NP [212]. In AF of IVDD patients, tears and cracks are commonly present, which may be caused by excessive ER stress in AF cells. Abnormal mechanical loading facilitated apoptosis of AF cells through nitric oxide-mediated ER stress [213]. Chen et al. [214] found that autophagy and apoptosis were mediated by ER stress in AF cells of mechanical loading-related IVDD model rats, suggesting that mechanical loading was involved in IVDD through crosstalk among ER stress, autophagy, and apoptosis in AF cells. Moreover, diabetes is a pivotal risk factor of IVDD, and the hyperglycemia is caused by diabetes induced apoptosis of AF cells through ER stress [215].

### ER stress in CEP degeneration

As discussed above, CEP is the main blood supply to IVD. Degeneration of CEP can severely hinder blood and nutrient supply to IVD, and then lead to IVDD [216]. Aberrant endplate chondrocyte apoptosis and calcification are two principal alterations in CEP degeneration [217]. ER stress may be an underlying mechanism leading to apoptosis and calcification of endplate chondrocytes; however, ER stress has not yet been linked to CEP degeneration.

## ER stress in the pathogenesis of SP

**Fig. 5:**
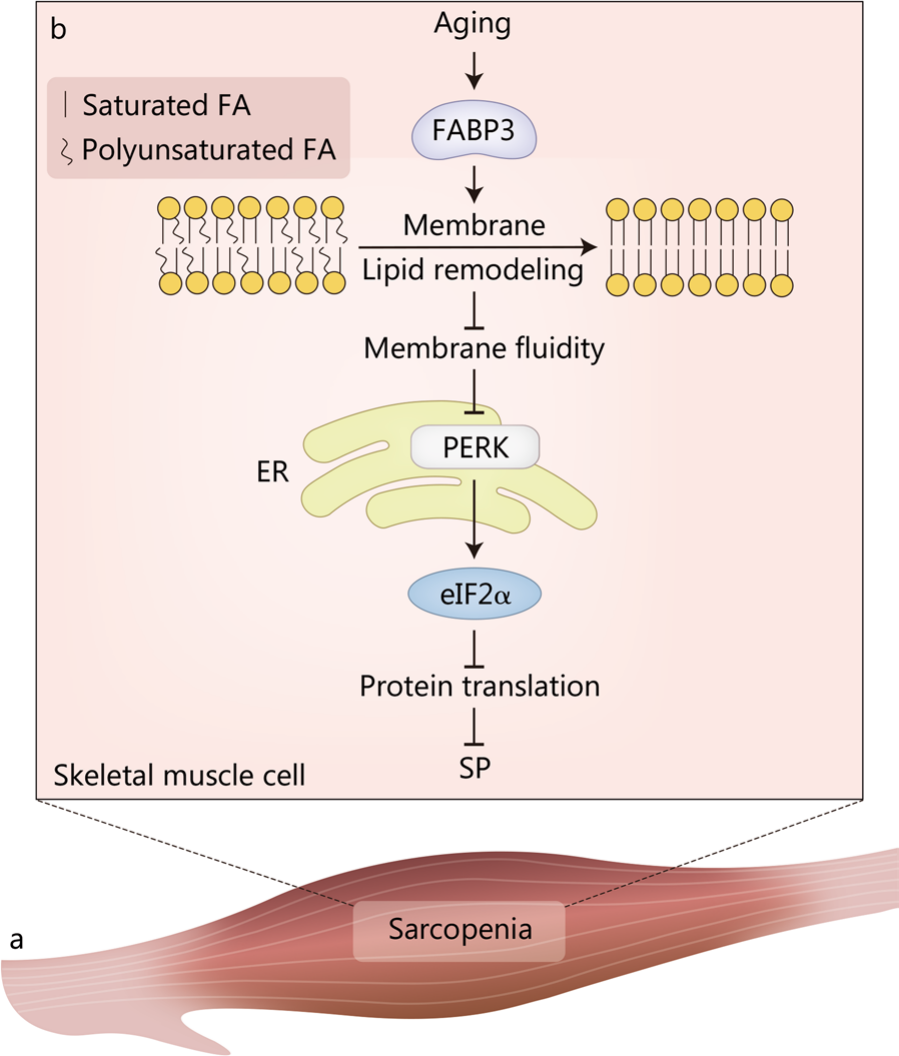
ER stress in the pathogenesis of sarcopenia (SP). **a** Schematic illustration of SP. **b** FABP3-dependent membrane lipid remodeling and decreased membrane fluidity result in ER stress and inhibit protein translation through the PERK-eIF2α pathway, ultimately leading to SP. eIF2α α-subunit of eukaryotic translation initiation factor 2, ER endoplasmic reticulum, FA fatty acid, FABP3 fatty acid binding protein 3, PERK protein kinase R-like ER kinase

The term SP, first coined by Irwin Rosenberg in 1989, comes from the Greek “sarx”, meaning flesh, and “penia”, meaning loss [218] (Fig. 5a). SP is a new disease that was recognized as an independent disease in 2016 by the International Classification of Diseases-10 code [219]. In 2010, the European Working Group on Sarcopenia in Older People (EWGSOP) published a definition of SP based solely on low muscle mass [220]. Subsequently, EWGSOP2 added low muscle strength as a primary criterion for SP to the new definition developed in 2018, as muscle strength is a more accurate predictor of adverse clinical outcomes in SP, such as falls, physical functional decline, frailty, impaired quality of life, increased health care costs, and mortality [5, 221]. At the same time, EWGSOP2 proposed a stepwise diagnostic method for SP by comprehensively measuring muscle mass, muscle strength, and physical performance [5]. According to the study, among the elderly hospitalized, hospitalization costs were more than five times higher for those with SP at the time of admission than for those without SP [222]. Unfortunately, there are still limited reports on the pathophysiology of SP. The heavy clinical burdens of SP prompt us to explore the pathogenesis of SP in depth. Etiologies of SP include neurological disorders, hormonal disturbances, nutritional disorders, immune factors, redox status of skeletal muscle, and reduced physical activity [223, 224]. From a cellular and molecular point of view, a mismatch in protein synthesis and degradation is the predominant factor contributing to SP [225]. Furthermore, a specialized ER network, called the sarcoplasmic reticulum, exists in skeletal muscle cells, and Ca^2+^ released from the sarcoplasmic reticulum plays a critical role in the process of skeletal muscle contraction [226]. ER stress may accelerate SP progression by affecting protein metabolism and Ca^2+^ release in skeletal muscle cells. It has been reported that the expression of ER stress biomarkers (ATF6, PERK, and IRE1) was significantly upregulated in SP skeletal muscle of lung cancer patients with cachexia [227]. Indoxol sulfate, a uremic toxin of chronic kidney disease, was used to construct a model of premature aging to study the pathogenesis of SP [225]. XBP1 and phosphorylated eIF2α played a pro-myogenic and anti-myogenic role in the differentiation of mouse myoblast C2C12 cells treated with indoxyl sulfate, respectively [225].

During aging, some critical molecules of the UPR, such as chaperones and enzymes, exhibit decreased expression and activity, leading to enhanced ER stress [228]. The expression of chaperones, including endoplasmic reticulum protein 29, 70 kD heat shock protein, and calreticulin, was decreased in skeletal muscle of 32-month-old rats compared with 6-month-old rats, whereas the expression levels of ER stress and apoptosis markers were increased [229]. Aging-related impairment of mitochondrial function and integrity could also lead to cellular oxidative stress, which then triggered ER stress in C2C12 myotubes [230]. On the other hand, aging-caused decrease in autophagy aggravated ER stress-induced loss of muscle mass [231]. Muscle RING Finger 1 is a muscle-specific E3 ubiquitin ligase whose transcription is increased in response to various stimuli inducing muscle loss [232]. Muscle RING Finger 1 has been reported to reduce muscle mass in aging mice by upregulating CHOP expression and inhibiting proteasome activity [233]. Fatty acid binding protein 3 (FABP3) is highly expressed in aged muscle and is considered a lipid chaperone that regulates fatty acid solubility, mobility, and utilization [234]. FABP3 overexpression in young muscle remodeled membrane lipid composition to that of senescent muscle and reduced membrane fluidity by replacing unsaturated FAs with saturated FAs [235]. FABP3-dependent membrane lipid remodeling and decreased membrane fluidity resulted in ER stress and inhibited protein translation through the PERK-eIF2α pathway, ultimately leading to SP (Fig. 5b). In sedentary and inactive individuals, optic atrophy 1 (OPA1), which is responsible for the fusion of the outer and inner mitochondrial membranes, showed an age-related decline, which was associated with the onset and progression of SP [236]. Ablation of *Opa1* resulted in ER stress, which induced catabolic processes and mass loss in skeletal muscle through UPR and forkhead box transcription factors [236]. Older adults are less responsive to anabolic stimuli, known as anabolic resistance, and may play an important role in the pathogenesis of SP [237–239]. Decreased phosphorylation of protein kinase B induced by ER stress led to low phosphorylation of proline-rich Akt substrate of 40 kD and allosteric inhibition of mTORC1 [240]. The mTORC1 pathway is indispensable in response to anabolic stimuli, and its inhibition is one of the mechanisms involved in anabolic resistance in skeletal muscle during aging [218]. Moreover, it is noteworthy that the loss of muscle strength is greater than the loss of muscle mass in older individuals [241]. Reduced Ca^2+^-releasing capacity and increased markers of ER stress have been found in aged sarcoplasmic reticulum [242, 243]. Therefore, ER stress-induced impairment of sarcoplasmic reticulum Ca^2+^ release may be another possible reason for the inhibition of muscle strength during SP.

Obesity is known to increase the risk of mortality in patients with SP [244]. Bryner et al. [245] revealed that palmitate mediated ER stress and apoptosis in C2C12 cells. Alleviation of ER stress could ameliorate palmitate-induced apoptosis in myotubes [246]. Excessive intake of lipids in mice was reported to cause ER stress in skeletal muscle [247]. However, the molecular processes by which obesity triggers ER stress remain unknown.

## Treatments regulating ER stress for degenerative musculoskeletal diseases

There are currently no effective and safe treatments to delay or halt the progression of degenerative musculoskeletal diseases, making them an unmet medical need and a clinical problem urgently required to be addressed. Nevertheless, the severe socioeconomic burden of degenerative musculoskeletal diseases and the desire of patients for health and longevity call for effective and safe therapeutic approaches. Excessive ER stress is one of the causative factors involving in the pathogenesis of degenerative musculoskeletal diseases. Hence, regulation of ER stress may be a potential and prospective therapeutic strategy to alleviate degenerative musculoskeletal diseases. Thereinafter, we will provide a systematic summary of therapeutic tactics modulating ER stress from two main perspectives: decreasing the production of misfolded and unfolded proteins (including assisting protein folding and blocking protein synthesis) and facilitating the degradation of misfolded and unfolded proteins (Table 1). Unfortunately, ER stress modulators are not currently in clinical trials for the treatment of degenerative musculoskeletal diseases, even though there have been clinical trials validating the efficacy and safety of ER stress modulators for the treatment of neurodegenerative diseases, such as Alzheimer’s disease and Parkinson’s disease (NCT03533257, NCT02046434). Furthermore, possible hurdles in the journey from bench to beside are discussed as well.

**Table 1:** Therapeutic reagents targeting ER stress to alleviate degenerative musculoskeletal diseases

Therapeutic reagents	Effects in targeting ER stress	Roles in degenerative musculoskeletal diseases	Level of evidence	References
4-PBA	Assist protein folding	Inhibit ER stress, apoptosis, and cartilage degradation in anterior cruciate ligament transection rat models Inhibit ER stress-mediated osteoclast differentiation in IL-1β-treated BMSCs Protect NP cell from AGEs-induced apoptosis	In vivo In vitro In vitro	[248] [129] [206]
TUDCA	Assist protein folding	Recover cell proliferation, reduce apoptosis and increase expression of type II collagen in OA chondrocyte Alleviate compression-induced apoptosis and necroptosis of NP cells	In vitro In vitro	[249] [250]
Salubrinal	Inhibit eIF2α dephosphorylation Inhibit protein translation	Inhibit ER stress-mediated upregulation of MMP13 Stimulate the osteoblastogenesis of MC3T3 E1 and inhibit the osteoclastogenesis of RAW264.7	In vitro In vitro	[251] [252]
Guanabenz	Inhibit eIF2α dephosphorylation Inhibit protein translation	Stimulate the osteoblastogenesis of MC3T3 E1 and inhibit the osteoclastogenesis of RAW264.7	In vitro	[252]
CBZ	Increase autophagy	Reduce ER stress, improve hypertrophic chondrocyte differentiation, accelerate bone growth rate, and decrease skeletal dysplasia in metaphyseal chondrodysplasia type Schmid mouse model	In vivo	[253]

*ER* endoplasmic reticulum, *4-PBA* 4-phenylbutiric acid, *BMSCs* bone marrow mesenchymal stem cells, *NP* nucleus pulposus, *AGEs* advanced glycation end products, *TUDCA* tauroursodeoxycholic acid, *OA* osteoarthritis, *eIF2α* α-subunit of eukaryotic translation initiation factor 2, *MMP13* matrix metallopeptidase-13, *CBZ* carbamazepin

Chemical chaperones are reagents that regulate ER stress by assisting proper protein folding, such as 4-phenylbutiric acid (4-PBA) and tauroursodeoxycholic acid (TUDCA). 4-PBA, a small molecule chemical chaperone, was reported to suppress ER stress, apoptosis, and damage of cartilage in anterior cruciate ligament transection rats with OA [248]. Moreover, 4-PBA restrained ER stress-mediated osteoclast differentiation in IL-1β-treated BMSCs and protected NP cells from AGEs-induced apoptosis by inhibiting ER stress [129, 206]. Currently, 4-PBA is mainly recommended for clinical treatment of inherited urea cycle disorders; whereas, there are no clinical trials on 4-PBA for the treatment of degenerative musculoskeletal diseases [254]. TUDCA is another chemical chaperone playing a critical role in controlling ER stress. Compared with tunicamycin-treated chondrocytes, TUDCA-treated chondrocytes exhibited reduced levels of ER stress markers, recovered cell proliferation, decreased apoptosis, and increased expression of type II collagen [249]. Moreover, TUDCA alleviated compression-induced apoptosis and necroptosis of NP cells by reducing ER stress [250]. TUDCA has been approved by the Food and Drug Administration for primary biliary cholangitis therapy in modern clinical practice. Considering that TUDCA is transported into cells via the Na^+^/taurocholate co-transporting peptides mainly expressed on the membrane of hepatocytes, the clinical application of TUDCA in non-liver diseases such as degenerative musculoskeletal diseases may be somewhat confined by off-target effects [255]. Furthermore, blocking protein synthesis is another approach to avoid the accumulation of misfolded and unfolded proteins in the ER and subsequently regulate ER stress. Salubrinal is a selective inhibitor of eIF2α phosphatase that interdicts protein synthesis by increasing eIF2α phosphorylation and ultimately suppresses ER stress [256]. Hamamura et al. [251] found that salubrinal downregulated ER stress-mediated matrix metallopeptidase-13 expression by suppressing p38 MAPK in tunicamycin-treated chondrocytes. In addition, salubrinal and guanabenz (another selective inhibitor of eIF2α dephosphorylation) were demonstrated to not only stimulate the osteoblastogenesis of MC3T3 E1, but also inhibit the osteoclastogenesis of RAW264.7 [252]. Another strategy to manipulate ER stress is through degradation of misfolded and unfolded proteins accumulated in the ER, which is similar to ERAD. As a clinically approved autophagy stimulant used for the treatment of epileptic seizures and bipolar disorder, carbamazepine was testified to relieve ER stress and improve hypertrophic differentiation of chondrocytes in metaphyseal chondrodysplasia type Schmid mouse model, giving rise to an accelerated bone growth rate and decreased skeletal dysplasia [253]. However, it has not yet been determined, even in preclinical studies, whether carbamazepine can delay the progression of degenerative musculoskeletal diseases by mitigating ER stress.

With the vigorous development and expansion of translational medicine, it has always been the long-cherished desire of clinical and pharmaceutical workers to facilitate the achievements of basic and academic research to clinical application and marketing. Despite tremendous strides have been made in the regulation of ER stress for the treatment of degenerative musculoskeletal diseases, significant knowledge gap still lies in the bench-to-beside research and the journey to the clinic is not as smooth as expected. Firstly, more comprehensive and in-depth in vitro and in vivo experiments are required to expound the pathogenic and protective roles of ER stress in degenerative musculoskeletal diseases. As previously suggested, mild ER stress is protective, whereas excessive ER stress leads to disturbed ER homeostasis, cellular dysfunction, and even cell death. A better understanding of ER stress in the pathogenesis of degenerative musculoskeletal diseases is warranted for the development of pharmacological avenues to modulate ER stress for the prevention and treatment of degenerative musculoskeletal diseases. Secondly, the targets alternative to modulate ER stress are various, but the most appropriate ones for designing drugs remain to be identified. Thirdly, disease phenotypes in rodent models (generally referring to mice and rats) with degenerative musculoskeletal diseases are not always concordant with humans, thus potent modulators of ER stress in rodent models are not necessarily effective in patients. Because pathogenesis is more complicated and represents significant heterogeneity in humans, ER stress modulators that have been validated for efficacy and safety in vivo are not directly available for clinical trials. Further investigations of ER stress modulators in larger animal models, such as pigs and bovines, which are more similar to humans in terms of anatomy, body size, and physiological status, are needed. Next, reasonable drug concentrations, dosage forms, routes and frequency of administration for clinical trials have not been determined. Eventually, the poor selectivity of free ER stress modulators results in the susceptibility to aggregate in tissue or cells at non-disease sites, causing potential systemic side effects, decreased bioavailability, and reduced efficacy. TUDCA, for example, is transported into the cell via the Na^+^/taurocholate co-transporting peptides highly expressed on hepatocytes and is therefore prone to off-target effects. The use of drug delivery systems loaded with ER stress regulators allows for targeted, long-lasting and safe delivery of drugs to disease sites, which is a promising curative strategy.

## Conclusions

Degenerative musculoskeletal diseases (including OA, OP, IVDD, and SP) are becoming increasingly prevalent as the aging population increases worldwide. Degenerative musculoskeletal diseases not only increase the global health care burden, but also result in absenteeism owing to their symptoms and adverse clinical outcomes, which imposes a huge direct and indirect socioeconomic burden on families and society. Meanwhile, rapid economic and social development has promoted the desire of the elderly for health and longevity. However, our understanding of the pathogenesis of degenerative musculoskeletal diseases remains limited, hindering the advancement of effective and safe treatments. The imbalance between pursuing health and the low efficacy and safety of existing therapies provides a catalyst for the research of degenerative musculoskeletal diseases. Through sophisticated molecular mechanisms, ER stress is able to influence cartilage degeneration, synovitis, meniscal lesion, subchondral bone remodeling of OA, bone remodeling and angiogenesis of OP, NP degeneration, AF rupture, CEP degeneration of IVDD, and SP, contributing to degenerative musculoskeletal diseases. Regulation of ER stress can slow the progression of degenerative musculoskeletal diseases by regulating cartilage degeneration, bone remodeling, and NP degeneration to a certain extent in vitro or in vivo. Moreover, we have presented the possible future research directions of ER stress in degenerative musculoskeletal diseases, the latent challenges that will be encountered in the clinical translation of ER stress modulators, and the corresponding solutions. Although no ER stress modulators have yet entered clinical trials, these preliminary findings set the stage for further evaluation of the feasibility, efficacy, and safety of modulating ER stress for the treatment of human musculoskeletal degenerative diseases.

## Data Availability

Not applicable.

## Abbreviations

AF: Annulus fibrosus
AGEs: Advanced glycation end products
Akt: Protein kinase B
ATF4: Activating transcription factor 4
ATF6: Activating transcription factor 6
BiP: Binding immunoglobulin protein
BMSCs: Bone marrow mesenchymal stem cells
Ca^2+^: Calcium
CEP: Cartilaginous endplate
CHOP: C/EBP homologous protein
C/EBPβ: CCAAT/enhancer binding protein β
ECM: Extracellular matrix
eIF2α: α-subunit of eukaryotic translation initiation factor 2
ER: Endoplasmic reticulum
ERAD: ER-associated degradation
EWGSOP: European Working Group on Sarcopenia in Older People
FABP3: Fatty acid binding protein 3
FAs: Fatty acids
FLSs: Fibroblast-like synoviocytes
GLP-1: Glucagon-like peptide-1
GLUT1: Glucose transporter 1
GRP78: Glycoprotein 78 kD glucose-regulated protein
HIF-1α: Hypoxia-inducible factor-1α
IGF-1: Insulin-like growth factor-1
IL: Interleukin
IRE1: Inositol-requiring enzyme 1
IVD: Intervertebral disc
IVDD: Intervertebral disc degeneration
JNK: c-Jun N-terminal kinase
MAPK: Mitogen-activated protein kinase
mTORC1: Mechanistic target of rapamycin complex 1
NFATc1: Nuclear factor of activated T cells cytoplasmic 1
NF-κB: Nuclear factor-kappa B
NLRP3: NOD-like receptor family pyrin domain containing 3
NP: Nucleus pulposus
OA: Osteoarthritis
OP: Osteoporosis
PERK: Protein kinase R-like ER kinase
RANKL: Receptor activator of NF-κB ligand
ROS: Reactive oxygen species
Runx2: Runt-related transcription factor 2
SIRT1: Sirtuin1
Smad: Small mother against decapentaplegic
SP: Sarcopenia
SREBP: Sterol response element binding protein
TUDCA: Tauroursodeoxycholic acid
UPR: Unfolded protein response
XBP-1: X-binding protein 1
4-PBA: 4-phenylbutiric acid

## References

[1] Zheng YL, Song G, Guo JB, Su X, Chen YM, Yang Z (2021). Interactions among lncRNA/circRNA, miRNA, and mRNA in musculoskeletal degenerative diseases. Front Cell Dev Biol.

[2] Glyn-Jones S, Palmer AJR, Agricola R, Price AJ, Vincent TL, Weinans H (2015). Osteoarthr Lancet.

[3] Lane NE (2006). Epidemiology, etiology, and diagnosis of osteoporosis. Am J Obstet Gynecol.

[4] Risbud MV, Shapiro IM (2014). Role of cytokines in intervertebral disc degeneration: pain and disc content. Nat Rev Rheumatol.

[5] Cruz-Jentoft AJ, Sayer AA, Sarcopenia (2019). Lancet.

[6] Rellmann Y, Eidhof E, Dreier R, Review (2021). ER stress-induced cell death in osteoarthritic cartilage. Cell Signal.

[7] Black DM, Rosen CJ (2016). Clinical practice. Postmenopausal osteoporosis. N Engl J Med.

[8] Francisco V, Pino J, Gonzalez-Gay MA, Lago F, Karppinen J, Tervonen O (2022). A new immunometabolic perspective of intervertebral disc degeneration. Nat Rev Rheumatol.

[9] Song Y, Wu Z, Zhao P (2022). The function of metformin in aging-related musculoskeletal disorders. Front Pharmacol.

[10] Oakes SA, Papa FR (2015). The role of endoplasmic reticulum stress in human pathology. Annu Rev Pathol.

[11] Tabas I, Ron D (2011). Integrating the mechanisms of apoptosis induced by endoplasmic reticulum stress. Nat Cell Biol.

[12] Krebs J, Agellon LB, Michalak M (2015). Ca^2+^ homeostasis and endoplasmic reticulum (ER) stress: an integrated view of calcium signaling. Biochem Biophys Res Commun.

[13] Sano R, Reed JC (2013). ER stress-induced cell death mechanisms. Biochim Biophys Acta.

[14] Rahmati M, Moosavi MA, McDermott MF (2018). ER stress: a therapeutic target in rheumatoid arthritis?. Trends Pharmacol Sci.

[15] Hong J, Kim K, Kim JH, Park Y (2017). The role of endoplasmic reticulum stress in cardiovascular disease and exercise. Int J Vasc Med.

[16] Yong J, Johnson JD, Arvan P, Han J, Kaufman RJ (2021). Therapeutic opportunities for pancreatic β-cell ER stress in diabetes mellitus. Nat Rev Endocrinol.

[17] Cubillos-Ruiz JR, Bettigole SE, Glimcher LH (2017). Tumorigenic and immunosuppressive effects of endoplasmic reticulum stress in cancer. Cell.

[18] Walter P, Ron D (2011). The unfolded protein response: from stress pathway to homeostatic regulation. Science.

[19] Lin JH, Walter P, Yen TS (2008). Endoplasmic reticulum stress in Disease pathogenesis. Annu Rev Pathol.

[20] Siwecka N, Rozpedek-Kaminska W, Wawrzynkiewicz A, Pytel D, Diehl JA, Majsterek I (2021). The structure, activation and signaling of IRE1 and its role in determining cell fate. Biomedicines.

[21] Kopp MC, Larburu N, Durairaj V, Adams CJ, Ali MMU (2019). UPR proteins IRE1 and PERK switch BiP from chaperone to ER stress sensor. Nat Struct Mol Biol.

[22] Kohno K, Normington K, Sambrook J, Gething MJ, Mori K (1993). The promoter region of the yeast KAR2 (BiP) gene contains a regulatory domain that responds to the presence of unfolded proteins in the endoplasmic reticulum. Mol Cell Biol.

[23] Ron D, Walter P (2007). Signal integration in the endoplasmic reticulum unfolded protein response. Nat Rev Mol Cell Biol.

[24] Ye J, Rawson RB, Komuro R, Chen X, Davé UP, Prywes R (2000). ER stress induces cleavage of membrane-bound ATF6 by the same proteases that process SREBPs. Mol Cell.

[25] Adachi Y, Yamamoto K, Okada T, Yoshida H, Harada A, Mori K (2008). ATF6 is a transcription factor specializing in the regulation of quality control proteins in the endoplasmic reticulum. Cell Struct Funct.

[26] Mohammad-Qureshi SS, Jennings MD, Pavitt GD (2008). Clues to the mechanism of action of eIF2B, the guanine-nucleotide-exchange factor for translation initiation. Biochem Soc Trans.

[27] Harding HP, Novoa I, Zhang Y, Zeng H, Wek R, Schapira M (2000). Regulated translation initiation controls stress-induced gene expression in mammalian cells. Mol Cell.

[28] Han J, Kaufman RJ (2017). Physiological/pathological ramifications of transcription factors in the unfolded protein response. Genes Dev.

[29] Morishima N, Nakanishi K, Nakano A (2011). Activating transcription factor-6 (ATF6) mediates apoptosis with reduction of myeloid cell Leukemia sequence 1 (Mcl-1) protein via induction of WW domain binding protein 1. J Biol Chem.

[30] Acosta-Alvear D, Karagöz GE, Fröhlich F, Li H, Walther TC, Walter P (2018). The unfolded protein response and endoplasmic reticulum protein targeting machineries converge on the stress sensor IRE1. Elife.

[31] Poothong J, Sopha P, Kaufman RJ, Tirasophon W (2010). Domain compatibility in Ire1 kinase is critical for the unfolded protein response. FEBS Lett.

[32] Karagöz GE, Acosta-Alvear D, Nguyen HT, Lee CP, Chu F, Walter P (2017). An unfolded protein-induced conformational switch activates mammalian IRE1. Elife.

[33] Maurel M, Chevet E, Tavernier J, Gerlo S (2014). Getting RIDD of RNA: IRE1 in cell fate regulation. Trends Biochem Sci.

[34] Coelho DS, Domingos PM (2014). Physiological roles of regulated Ire1 dependent decay. Front Genet.

[35] Sozen E, Yazgan B, Tok OE, Demirel T, Ercan F, Proto JD (2020). Cholesterol induced autophagy via IRE1/JNK pathway promotes autophagic cell death in heart tissue. Metabolism.

[36] Zheng N, Shabek N (2017). Ubiquitin ligases: structure, function, and regulation. Annu Rev Biochem.

[37] Bernales S, McDonald KL, Walter P (2006). Autophagy counterbalances endoplasmic reticulum expansion during the unfolded protein response. PLoS Biol.

[38] Sen R, Hurley JA, StatPearls (2023). Osteoarthritis.

[39] Kung LHW, Mullan L, Soul J, Wang P, Mori K, Bateman JF (2019). Cartilage endoplasmic reticulum stress may influence the onset but not the progression of experimental osteoarthritis. Arthritis Res Ther.

[40] Robinson WH, Lepus CM, Wang Q, Raghu H, Mao R, Lindstrom TM (2016). Low-grade inflammation as a key mediator of the pathogenesis of osteoarthritis. Nat Rev Rheumatol.

[41] Li YH, Tardif G, Hum D, Kapoor M, Fahmi H, Pelletier JP (2016). The unfolded protein response genes in human osteoarthritic chondrocytes: PERK emerges as a potential therapeutic target. Arthritis Res Ther.

[42] Haywood J, Yammani RR (2016). Free fatty acid palmitate activates unfolded protein response pathway and promotes apoptosis in meniscus cells. Osteoarthr Cartil.

[43] Lin Z, Teng C, Ni L, Zhang Z, Lu X, Lou J (2021). Echinacoside upregulates Sirt1 to suppress endoplasmic reticulum stress and inhibit extracellular matrix degradation in vitro and ameliorates osteoarthritis in vivo. Oxid Med Cell Longev.

[44] Hecht JT, Veerisetty AC, Wu J, Coustry F, Hossain MG, Chiu F (2021). Primary osteoarthritis early joint degeneration induced by endoplasmic reticulum stress is mitigated by resveratrol. Am J Pathol.

[45] Makris EA, Gomoll AH, Malizos KN, Hu JC, Athanasiou KA (2015). Repair and tissue engineering techniques for articular cartilage. Nat Rev Rheumatol.

[46] Bei HP, Hung PM, Yeung HL, Wang S, Zhao X (2021). Bone-a-petite: engineering exosomes towards bone, osteochondral, and cartilage repair. Small.

[47] Thomas CM, Fuller CJ, Whittles CE, Sharif M (2007). Chondrocyte death by apoptosis is associated with cartilage matrix degradation. Osteoarthr Cartil.

[48] Loeser RF (1997). Growth factor regulation of chondrocyte integrins. Differential effects of insulin-like growth factor 1 and transforming growth factor beta on alpha 1 beta 1 integrin expression and chondrocyte adhesion to type VI collagen. Arthritis Rheum.

[49] Cravero JD, Carlson CS, Im HJ, Yammani RR, Long D, Loeser RF (2009). Increased expression of the Akt/PKB inhibitor TRB3 in osteoarthritic chondrocytes inhibits insulin-like growth factor 1-mediated cell survival and proteoglycan synthesis. Arthritis Rheum.

[50] Nazli SA, Loeser RF, Chubinskaya S, Willey JS, Yammani RR (2017). High fat-diet and saturated fatty acid palmitate inhibits IGF-1 function in chondrocytes. Osteoarthr Cartil.

[51] Hamamura K, Goldring MB, Yokota H (2009). Involvement of p38 MAPK in regulation of MMP13 mRNA in chondrocytes in response to surviving stress to endoplasmic reticulum. Arch Oral Biol.

[52] Yu X, Xu X, Dong W, Yang C, Luo Y, He Y (2022). DDIT3/CHOP mediates the inhibitory effect of ER stress on chondrocyte differentiation by AMPKα-SIRT1 pathway. Biochim Biophys Acta Mol Cell Res.

[53] Wu H, Meng Z, Jiao Y, Ren Y, Yang X, Liu H (2020). The endoplasmic reticulum stress induced by tunicamycin affects the viability and autophagy activity of chondrocytes. J Clin Lab Anal.

[54] Yang H, Wen Y, Zhang M, Liu Q, Zhang H, Zhang J (2020). MTORC1 coordinates the autophagy and apoptosis signaling in articular chondrocytes in osteoarthritic temporomandibular joint. Autophagy.

[55] Li K, Yang P, Zhang Y, Zhang Y, Cao H, Liu P (2021). DEPTOR prevents osteoarthritis development via interplay with TRC8 to reduce endoplasmic reticulum stress in chondrocytes. J Bone Miner Res.

[56] Everett RD, Meredith M, Orr A, Cross A, Kathoria M, Parkinson J (1997). A novel ubiquitin-specific protease is dynamically associated with the PML nuclear domain and binds to a herpesvirus regulatory protein. EMBO J.

[57] Dong X, Yang C, Luo Y, Dong W, Xu X, Wu Y (2022). USP7 attenuates endoplasmic reticulum stress and NF-κB signaling to modulate chondrocyte proliferation, apoptosis, and inflammatory response under inflammation. Oxid Med Cell Longev.

[58] Coryell PR, Diekman BO, Loeser RF (2021). Mechanisms and therapeutic implications of cellular senescence in osteoarthritis. Nat Rev Rheumatol.

[59] Martel-Pelletier J, Barr AJ, Cicuttini FM, Conaghan PG, Cooper C, Goldring MB (2016). Osteoarthr Nat Rev Dis Primers.

[60] Chadwick SR, Lajoie P (2019). Endoplasmic reticulum stress coping mechanisms and lifespan regulation in health and diseases. Front Cell Dev Biol.

[61] Paz Gavilan M, Vela J, Castano A, Ramos B, del Rio JC, Vitorica J (2006). Cellular environment facilitates protein accumulation in aged rat hippocampus. Neurobiol Aging.

[62] Lee BY, Han JA, Im JS, Morrone A, Johung K, Goodwin EC (2006). Senescence-associated beta-galactosidase is lysosomal beta-galactosidase. Aging Cell.

[63] Hirose J, Yamabe S, Takada K, Okamoto N, Nagai R, Mizuta H (2011). Immunohistochemical distribution of advanced glycation end products (AGEs) in human osteoarthritic cartilage. Acta Histochem.

[64] Yamabe S, Hirose J, Uehara Y, Okada T, Okamoto N, Oka K (2013). Intracellular accumulation of advanced glycation end products induces apoptosis via endoplasmic reticulum stress in chondrocytes. FEBS J.

[65] Tan L, Register TC, Yammani RR (2020). Age-related decline in expression of molecular chaperones induces endoplasmic reticulum stress and chondrocyte apoptosis in articular cartilage. Aging Dis.

[66] Nuss JE, Choksi KB, DeFord JH, Papaconstantinou J (2008). Decreased enzyme activities of chaperones PDI and BiP in aged mouse livers. Biochem Biophys Res Commun.

[67] Liu Y, Zhu H, Yan X, Gu H, Gu Z, Liu F (2017). Endoplasmic reticulum stress participates in the progress of senescence and apoptosis of osteoarthritis chondrocytes. Biochem Biophys Res Commun.

[68] Ott C, Jacobs K, Haucke E, Navarrete Santos A, Grune T, Simm A (2014). Role of advanced glycation end products in cellular signaling. Redox Biol.

[69] Rasheed Z, Haqqi TM (2012). Endoplasmic reticulum stress induces the expression of COX-2 through activation of eIF2α, p38-MAPK and NF-κB in advanced glycation end products stimulated human chondrocytes. Biochim Biophys Acta.

[70] Nelson G, Wordsworth J, Wang C, Jurk D, Lawless C, Martin-Ruiz C (2012). A senescent cell bystander effect: senescence-induced senescence. Aging Cell.

[71] Opie LH, Walfish PG (1963). Plasma free fatty acid concentrations in obesity. N Engl J Med.

[72] Sandell LJ (2012). Etiology of osteoarthritis: genetics and synovial joint development. Nat Rev Rheumatol.

[73] Tan L, Harper L, McNulty MA, Carlson CS, Yammani RR (2020). High-fat diet induces endoplasmic reticulum stress to promote chondrocyte apoptosis in mouse knee joints. FASEB J.

[74] Tan L, Yammani RR (2019). Nupr1 regulates palmitate-induced apoptosis in human articular chondrocytes. Biosci Rep..

[75] Tan L, Harper LR, Armstrong A, Carlson CS, Yammani RR (2021). Dietary saturated fatty acid palmitate promotes cartilage lesions and activates the unfolded protein response pathway in mouse knee joints. PLoS One.

[76] Sandoval DA, D’Alessio DA (2015). Physiology of proglucagon peptides: role of glucagon and GLP-1 in health and disease. Physiol Rev.

[77] Chen J, Xie JJ, Shi KS, Gu YT, Wu CC, Xuan J (2018). Glucagon-like peptide-1 receptor regulates endoplasmic reticulum stress-induced apoptosis and the associated inflammatory response in chondrocytes and the progression of osteoarthritis in rat. Cell Death Dis.

[78] Katz JN, Arant KR, Loeser RF (2021). Diagnosis and treatment of hip and knee osteoarthritis: a review. JAMA.

[79] Wilson MG, Michet CJ, Ilstrup DM, Melton LJ (1990). Idiopathic symptomatic osteoarthritis of the hip and knee: a population-based incidence study. Mayo Clin Proc..

[80] Srikanth VK, Fryer JL, Zhai G, Winzenberg TM, Hosmer D, Jones G (2005). A meta-analysis of sex differences prevalence, incidence and severity of osteoarthritis. Osteoarthr Cartil.

[81] Dreier R, Ising T, Ramroth M, Rellmann Y (2022). Estradiol inhibits ER stress-induced apoptosis in chondrocytes and contributes to a reduced osteoarthritic cartilage degeneration in female mice. Front Cell Dev Biol.

[82] Falconer J, Murphy AN, Young SP, Clark AR, Tiziani S, Guma M (2018). Review: synovial cell metabolism and chronic inflammation in rheumatoid arthritis. Arthritis Rheumatol.

[83] Orr C, Vieira-Sousa E, Boyle DL, Buch MH, Buckley CD, Canete JD (2017). Synovial tissue research: a state-of-the-art review. Nat Rev Rheumatol.

[84] Scanzello CR, Goldring SR (2012). The role of synovitis in osteoarthritis pathogenesis. Bone.

[85] Mathiessen A, Conaghan PG (2017). Synovitis in osteoarthritis: current understanding with therapeutic implications. Arthritis Res Ther.

[86] Li Q, Wen Y, Wang L, Chen B, Chen J, Wang H (2021). Hyperglycemia-induced accumulation of advanced glycosylation end products in fibroblast-like synoviocytes promotes knee osteoarthritis. Exp Mol Med.

[87] Khan MI, Rath S, Adhami VM, Mukhtar H (2018). Hypoxia driven glycation: mechanisms and therapeutic opportunities. Semin Cancer Biol.

[88] Zhang L, Xing R, Huang Z, Zhang N, Zhang L, Li X (2019). Inhibition of synovial macrophage pyroptosis alleviates synovitis and fibrosis in knee osteoarthritis. Mediators Inflamm.

[89] Han CY, Rho HS, Kim A, Kim TH, Jang K, Jun DW (2018). FXR inhibits endoplasmic reticulum stress-induced NLRP3 inflammasome in hepatocytes and ameliorates liver injury. Cell Rep.

[90] Liu Z, Liao T, Yang N, Ding L, Li X, Wu P (2021). Interventional effects of the topical of Sanse Powder essential oils nanoemulsion on knee osteoarthritis in rats by targeting the ERS/TXNIP/NLRP3 signaling axis. Front Pharmacol.

[91] Lee CH, Chiang CF, Kuo FC, Su SC, Huang CL, Liu JS (2021). High-molecular-weight hyaluronic acid inhibits IL-1β-induced synovial inflammation and macrophage polarization through the GRP78-NF-κB signaling pathway. Int J Mol Sci.

[92] Makris EA, Hadidi P, Athanasiou KA (2011). The knee meniscus: structure-function, pathophysiology, current repair techniques, and prospects for regeneration. Biomaterials.

[93] Nishimuta JF, Levenston ME (2015). Meniscus is more susceptible than cartilage to catabolic and anti-anabolic effects of adipokines. Osteoarthr Cartil.

[94] Mallik A, Yammani RR (2018). Saturated fatty acid palmitate negatively regulates autophagy by promoting ATG5 protein degradation in meniscus cells. Biochem Biophys Res Commun.

[95] Madry H, van Dijk CN, Mueller-Gerbl M (2010). The basic science of the subchondral bone. Knee Surg Sports Traumatol Arthrosc.

[96] Burr DB, Gallant MA (2012). Bone remodelling in osteoarthritis. Nat Rev Rheumatol.

[97] Intema F, Hazewinkel HA, Gouwens D, Bijlsma JW, Weinans H, Lafeber FP (2010). In early OA, thinning of the subchondral plate is directly related to cartilage damage: results from a canine ACLT-meniscectomy model. Osteoarthr Cartil.

[98] Li G, Yin J, Gao J, Cheng TS, Pavlos NJ, Zhang C (2013). Subchondral bone in osteoarthritis: insight into risk factors and microstructural changes. Arthritis Res Ther.

[99] Goldring SR, Goldring MB (2016). Changes in the osteochondral unit during osteoarthritis: structure, function and cartilage-bone crosstalk. Nat Rev Rheumatol.

[100] Zhu X, Chan YT, Yung PSH, Tuan RS, Jiang Y (2020). Subchondral bone remodeling: a therapeutic target for osteoarthritis. Front Cell Dev Biol.

[101] Hu W, Chen Y, Dou C, Dong S (2021). Microenvironment in subchondral bone: predominant regulator for the treatment of osteoarthritis. Ann Rheum Dis.

[102] Wang Y, Zhang T, Xu Y, Chen R, Qu N, Zhang B (2022). Suppressing phosphoinositide-specific phospholipases Cgamma1 promotes mineralization of osteoarthritic subchondral bone osteoblasts via increasing autophagy, thereby ameliorating articular cartilage degeneration. Bone.

[103] Guo J, Ren R, Sun K, Yao X, Lin J, Wang G (2020). PERK controls bone homeostasis through the regulation of osteoclast differentiation and function. Cell Death Dis.

[104] Wang Y, Han B, Ding J, Qiu C, Wang W (2020). Endoplasmic reticulum stress mediates osteocyte death under oxygen-glucose deprivation in vitro. Acta Histochem.

[105] Jilka RL, O’Brien CA (2016). The role of osteocytes in age-related bone loss. Curr Osteoporos Rep.

[106] Sun Y, Yuan Y, Wu W, Lei L, Zhang L (2021). The effects of locomotion on bone marrow mesenchymal stem cell fate: insight into mechanical regulation and bone formation. Cell Biosci.

[107] Zheng J, Gao Y, Lin H, Yuan C, Keqianzhi (2021). Enhanced autophagy suppresses inflammation-mediated bone loss through ROCK1 signaling in bone marrow mesenchymal stem cells. Cells Dev.

[108] Schulze-Tanzil G (2019). Intraarticular ligament degeneration is interrelated with cartilage and bone destruction in osteoarthritis. Cells.

[109] Kim HA, Kim I, Song YW, Kim DH, Niu J, Guermazi A (2011). The association between meniscal and cruciate ligament damage and knee pain in community residents. Osteoarthr Cartil.

[110] Li QX, Li ZY, Liu L, Ni QB, Yang X, Chen B (2020). Dexamethasone causes calcium deposition and degeneration in human anterior cruciate ligament cells through endoplasmic reticulum stress. Biochem Pharmacol.

[111] Shi L, Miao J, Chen D, Shi J, Chen Y (2019). Endoplasmic reticulum stress regulates mechanical stress-induced ossification of posterior longitudinal ligament. Eur Spine J.

[112] Krishnasamy P, Hall M, Robbins SR (2018). The role of skeletal muscle in the pathophysiology and management of knee osteoarthritis. Rheumatology (Oxford).

[113] Kim TJ, Lee HJ, Pyun DH, Abd El-Aty AM, Jeong JH, Jung TW (2021). Valdecoxib improves lipid-induced skeletal muscle insulin resistance via simultaneous suppression of inflammation and endoplasmic reticulum stress. Biochem Pharmacol.

[114] Hunter DJ, Bierma-Zeinstra S, Osteoarthritis (2019). Lancet.

[115] Fu K, Robbins SR, McDougall JJ (2018). Osteoarthritis: the genesis of pain. Rheumatology (Oxford).

[116] Inceoglu B, Bettaieb A, Trindade da Silva CA, Lee KS, Haj FG, Hammock BD (2015). Endoplasmic reticulum stress in the peripheral nervous system is a significant driver of neuropathic pain. Proc Natl Acad Sci U S A.

[117] Mao Y, Wang C, Tian X, Huang Y, Zhang Y, Wu H (2020). Endoplasmic reticulum stress contributes to nociception via neuroinflammation in a murine Bone cancer pain model. Anesthesiology.

[118] Wright NC, Looker AC, Saag KG, Curtis JR, Delzell ES, Randall S (2014). The recent prevalence of osteoporosis and low bone mass in the United States based on bone mineral density at the femoral neck or lumbar spine. J Bone Miner Res.

[119] Armas LA, Recker RR (2012). Pathophysiology of osteoporosis: new mechanistic insights. Endocrinol Metab Clin North Am.

[120] Li J, Yang S, Li X, Liu D, Wang Z, Guo J (2017). Role of endoplasmic reticulum stress in disuse osteoporosis. Bone.

[121] Yu H, Pardoll D, Jove R (2009). STATs in cancer inflammation and immunity: a leading role for STAT3. Nat Rev Cancer.

[122] Zhuang J, Gao R, Wu H, Wu X, Pan F (2015). Signal transducer and activator of transcription 3 regulates CCAAT-enhancer-binding homologous protein expression in osteoblasts through upregulation of microRNA-205. Exp Ther Med.

[123] Han C, Xie K, Yang C, Zhang F, Liang Q, Lan C (2021). HA15 alleviates bone loss in ovariectomy-induced osteoporosis by targeting HSPA5. Exp Cell Res.

[124] D’Autréaux B, Toledano MB (2007). ROS as signalling molecules: mechanisms that generate specificity in ROS homeostasis. Nat Rev Mol Cell Biol.

[125] Hu X, Li B, Wu F, Liu X, Liu M, Wang C (2021). GPX7 facilitates BMSCs osteoblastogenesis via ER stress and mTOR pathway. J Cell Mol Med.

[126] Kim JH, Kim K, Kim I, Seong S, Nam KI, Kim KK (2018). Endoplasmic reticulum-bound transcription factor CREBH stimulates RANKL-induced osteoclastogenesis. J Immunol.

[127] Yamashita T, Yao Z, Li F, Zhang Q, Badell IR, Schwarz EM (2007). NF-kappaB p50 and p52 regulate receptor activator of NF-kappaB ligand (RANKL) and Tumor necrosis factor-induced osteoclast precursor differentiation by activating c-Fos and NFATc1. J Biol Chem.

[128] Asagiri M, Sato K, Usami T, Ochi S, Nishina H, Yoshida H (2005). Autoamplification of NFATc1 expression determines its essential role in bone homeostasis. J Exp Med.

[129] Lee EG, Sung MS, Yoo HG, Chae HJ, Kim HR, Yoo WH (2014). Increased RANKL-mediated osteoclastogenesis by interleukin-1β and endoplasmic reticulum stress. Joint Bone Spine.

[130] Zhang K, Shen X, Wu J, Sakaki K, Saunders T, Rutkowski DT (2006). Endoplasmic reticulum stress activates cleavage of CREBH to induce a systemic inflammatory response. Cell.

[131] Yang YH, Li B, Zheng XF, Chen JW, Chen K, Jiang SD (2014). Oxidative damage to osteoblasts can be alleviated by early autophagy through the endoplasmic reticulum stress pathway–implications for the treatment of osteoporosis. Free Radic Biol Med.

[132] Collier JJ, Suomi F, Oláhová M, McWilliams TG, Taylor RW (2021). Emerging roles of ATG7 in human health and disease. EMBO Mol Med.

[133] Li H, Li D, Ma Z, Qian Z, Kang X, Jin X (2018). Defective autophagy in osteoblasts induces endoplasmic reticulum stress and causes remarkable bone loss. Autophagy.

[134] Wu M, Chen G, Li YP (2016). TGF-β and BMP signaling in osteoblast, skeletal development, and bone formation, homeostasis and disease. Bone Res.

[135] Zheng Z, Zhang X, Huang B, Liu J, Wei X, Shan Z (2021). Site-1 protease controls osteoclastogenesis by mediating LC3 transcription. Cell Death Differ.

[136] Briot K, Roux C, Thomas T, Blain H, Buchon D, Chapurlat R (2018). 2018 update of French recommendations on the management of postmenopausal osteoporosis. Joint Bone Spine.

[137] Suzuki R, Fujiwara Y, Saito M, Arakawa S, Shirakawa JI, Yamanaka M (2020). Intracellular accumulation of advanced glycation end products induces osteoblast apoptosis via endoplasmic reticulum stress. J Bone Miner Res.

[138] Guo YS, Sun Z, Ma J, Cui W, Gao B, Zhang HY (2014). 17β-estradiol inhibits ER stress-induced apoptosis through promotion of TFII-I-dependent Grp78 induction in osteoblasts. Lab Invest.

[139] Chotiyarnwong P, McCloskey EV (2020). Pathogenesis of glucocorticoid-induced osteoporosis and options for treatment. Nat Rev Endocrinol.

[140] Sato AY, Tu X, McAndrews KA, Plotkin LI, Bellido T (2015). Prevention of glucocorticoid induced-apoptosis of osteoblasts and osteocytes by protecting against endoplasmic reticulum (ER) stress in vitro and in vivo in female mice. Bone.

[141] Guo Y, Hao D, Hu H (2021). High doses of dexamethasone induce endoplasmic reticulum stress-mediated apoptosis by promoting calcium ion influx-dependent CHOP expression in osteoblasts. Mol Biol Rep.

[142] Mohsin S, Baniyas MM, AlDarmaki RS, Tekes K, Kalász H, Adeghate EA (2019). An update on therapies for the treatment of diabetes-induced osteoporosis. Expert Opin Biol Ther.

[143] Liu W, Zhu X, Wang Q, Wang L (2013). Hyperglycemia induces endoplasmic reticulum stress-dependent CHOP expression in osteoblasts. Exp Ther Med.

[144] Aguirre L, Napoli N, Waters D, Qualls C, Villareal DT, Armamento-Villareal R (2014). Increasing adiposity is associated with higher adipokine levels and lower bone mineral density in obese older adults. J Clin Endocrinol Metab.

[145] Jing L, Jia XW (2018). Lycium barbarum polysaccharide arbitrates palmitate-induced apoptosis in MC3T3E1 cells through decreasing the activation of ERS-mediated apoptosis pathway. Mol Med Rep.

[146] Gillet C, Spruyt D, Rigutto S, Dalla Valle A, Berlier J, Louis C (2015). Oleate abrogates palmitate-induced lipotoxicity and proinflammatory response in human bone marrow-derived mesenchymal stem cells and osteoblastic cells. Endocrinology.

[147] Park SJ, Kim KJ, Kim WU, Oh IH, Cho CS (2012). Involvement of endoplasmic reticulum stress in homocysteine-induced apoptosis of osteoblastic cells. J Bone Miner Metab.

[148] Ozcivici E, Luu YK, Adler B, Qin YX, Rubin J, Judex S (2010). Mechanical signals as anabolic agents in bone. Nat Rev Rheumatol.

[149] Styner M, Meyer MB, Galior K, Case N, Xie Z, Sen B (2012). Mechanical strain downregulates C/EBPβ in MSC and decreases endoplasmic reticulum stress. PLoS ONE.

[150] Chalil S, Jaspers RT, Manders RJ, Klein-Nulend J, Bakker AD, Deldicque L (2015). Increased endoplasmic reticulum stress in mouse osteocytes with aging alters Cox-2 response to mechanical stimuli. Calcif Tissue Int.

[151] Munoz J, Akhavan NS, Mullins AP, Arjmandi BH (2020). Macrophage polarization and osteoporosis: a review. Nutrients..

[152] Fischer V, Haffner-Luntzer M (2022). Interaction between bone and immune cells: implications for postmenopausal osteoporosis. Semin Cell Dev Biol.

[153] Brown BN, Ratner BD, Goodman SB, Amar S, Badylak SF (2012). Macrophage polarization: an opportunity for improved outcomes in biomaterials and regenerative medicine. Biomaterials.

[154] Mantovani A, Sica A, Sozzani S, Allavena P, Vecchi A, Locati M (2004). The chemokine system in diverse forms of macrophage activation and polarization. Trends Immunol.

[155] Murray Peter J, Allen Judith E, Biswas Subhra K, Fisher Edward A, Gilroy Derek W, Goerdt S (2014). Macrophage activation and polarization: nomenclature and experimental guidelines. Immunity.

[156] Loi F, Córdova LA, Zhang R, Pajarinen J, Lin TH, Goodman SB (2016). The effects of immunomodulation by macrophage subsets on osteogenesis in vitro. Stem Cell Res Ther.

[157] Gong L, Zhao Y, Zhang Y, Ruan Z (2016). The macrophage polarization regulates MSC osteoblast differentiation in vitro. Ann Clin Lab Sci.

[158] Yuan Y, Jiao P, Wang Z, Chen M, Du H, Xu L (2022). Endoplasmic reticulum stress promotes the release of exosomal PD-L1 from Head and Neck cancer cells and facilitates M2 macrophage polarization. Cell Communication and Signaling.

[159] Lu C, Shi W, Hu W, Zhao Y, Zhao X, Dong F (2022). Endoplasmic reticulum stress promotes Breast cancer cells to release exosomes circ_0001142 and induces M2 polarization of macrophages to regulate Tumor progression. Pharmacol Res.

[160] Du N, Wu K, Zhang J, Wang L, Pan X, Zhu Y (2021). Inonotsuoxide B regulates M1 to M2 macrophage polarization through sirtuin-1/endoplasmic reticulum stress axis. Int Immunopharmacol.

[161] Manz MG, Boettcher S (2014). Emergency granulopoiesis. Nat Rev Immunol.

[162] Poubelle PE, Chakravarti A, Fernandes MJ, Doiron K, Marceau AA (2007). Differential expression of RANK, RANK-L, and osteoprotegerin by synovial fluid neutrophils from patients with rheumatoid arthritis and by healthy human blood neutrophils. Arthritis Res Ther.

[163] Chakravarti A, Raquil MA, Tessier P, Poubelle PE (2009). Surface RANKL of toll-like receptor 4-stimulated human neutrophils activates osteoclastic bone resorption. Blood.

[164] Hu X, Sun Y, Xu W, Lin T, Zeng H (2017). Expression of RANKL by peripheral neutrophils and its association with bone mineral density in COPD. Respirology.

[165] Sule G, Abuaita BH, Steffes PA, Fernandes AT, Estes SK, Dobry C (2021). Endoplasmic reticulum stress sensor IRE1α propels neutrophil hyperactivity in lupus. J Clin Invest.

[166] Filipowska J, Tomaszewski KA, Niedźwiedzki Ł, Walocha JA, Niedźwiedzki T (2017). The role of vasculature in bone development, regeneration and proper systemic functioning. Angiogenesis.

[167] Shangguan WJ, Zhang YH, Li ZC, Tang LM, Shao J, Li H (2017). Naringin inhibits vascular endothelial cell apoptosis via endoplasmic reticulum stress and mitochondrialmediated pathways and promotes intraosseous angiogenesis in ovariectomized rats. Int J Mol Med.

[168] Kavanagh KL, Guo K, Dunford JE, Wu X, Knapp S, Ebetino FH (2006). The molecular mechanism of nitrogen-containing bisphosphonates as antiosteoporosis drugs. Proc Natl Acad Sci U S A.

[169] Kusumbe AP, Adams RH (2014). Osteoclast progenitors promote bone vascularization and osteogenesis. Nat Med.

[170] Ding N, Liu C, Yao L, Bai Y, Cheng P, Li Z (2018). Alendronate induces osteoclast precursor apoptosis via peroxisomal dysfunction mediated ER stress. J Cell Physiol.

[171] Kamali A, Ziadlou R, Lang G, Pfannkuche J, Cui S, Li Z (2021). Small molecule-based treatment approaches for intervertebral disc degeneration: current options and future directions. Theranostics.

[172] Wang D, He X, Zheng C, Wang C, Peng P, Gao C (2021). Endoplasmic reticulum stress: an emerging therapeutic target for intervertebral disc degeneration. Front Cell Dev Biol.

[173] Hartvigsen J, Hancock MJ, Kongsted A, Louw Q, Ferreira ML, Genevay S (2018). What low back pain is and why we need to pay attention. Lancet.

[174] Ehrlich GE (2003). Low back pain. Bull World Health Organ.

[175] Vergroesen PP, Kingma I, Emanuel KS, Hoogendoorn RJ, Welting TJ, van Royen BJ (2015). Mechanics and biology in intervertebral disc degeneration: a vicious circle. Osteoarthr Cartil.

[176] Bian Q, Ma L, Jain A, Crane JL, Kebaish K, Wan M (2017). Mechanosignaling activation of TGFβ maintains intervertebral disc homeostasis. Bone Res.

[177] Zhao CQ, Zhang YH, Jiang SD, Jiang LS, Dai LY (2010). Both endoplasmic reticulum and mitochondria are involved in disc cell apoptosis and intervertebral disc degeneration in rats. Age (Dordr).

[178] Liao Z, Luo R, Li G, Song Y, Zhan S, Zhao K (2019). Exosomes from mesenchymal stem cells modulate endoplasmic reticulum stress to protect against nucleus pulposus cell death and ameliorate intervertebral disc degeneration in vivo. Theranostics.

[179] Kang H, Dong Y, Peng R, Liu H, Guo Q, Song K (2022). Inhibition of IRE1 suppresses the catabolic effect of IL-1β on nucleus pulposus cell and prevents intervertebral disc degeneration in vivo. Biochem Pharmacol.

[180] Ling Z, Liu Y, Wang Z, Zhang Z, Chen B, Yang J (2021). Single-cell RNA-seq analysis reveals macrophage involved in the progression of human intervertebral disc degeneration. Front Cell Dev Biol.

[181] Rodrigues-Pinto R, Richardson SM, Hoyland JA (2014). An understanding of intervertebral disc development, maturation and cell phenotype provides clues to direct cell-based tissue regeneration therapies for disc degeneration. Eur Spine J.

[182] Zhong H, Zhou Z, Guo L, Liu F, Zheng B, Bi S (2021). The miR-623/CXCL12 axis inhibits LPS-induced nucleus pulposus cell apoptosis and senescence. Mech Ageing Dev.

[183] Gawri R, Rosenzweig DH, Krock E, Ouellet JA, Stone LS, Quinn TM (2014). High mechanical strain of primary intervertebral disc cells promotes secretion of inflammatory factors associated with disc degeneration and pain. Arthritis Res Ther.

[184] Wang C, Gonzales S, Levene H, Gu W, Huang CY (2013). Energy metabolism of intervertebral disc under mechanical loading. J Orthop Res.

[185] Xiang H, Su W, Wu X, Chen W, Cong W, Yang S (2020). Exosomes derived from human urine-derived stem cells inhibit intervertebral disc degeneration by ameliorating endoplasmic reticulum stress. Oxid Med Cell Longev.

[186] Cao J, Zhang Y, Wang T, Li B (2018). Endoplasmic reticulum stress is involved in baicalin protection on chondrocytes from patients with osteoarthritis. Dose Response.

[187] Vandenabeele P, Galluzzi L, Vanden Berghe T, Kroemer G (2010). Molecular mechanisms of necroptosis: an ordered cellular Explosion. Nat Rev Mol Cell Biol.

[188] Lin H, Peng Y, Li J, Wang Z, Chen S, Qing X (2021). Reactive oxygen species regulate endoplasmic reticulum stress and ER-mitochondrial Ca^2+^ crosstalk to promote programmed necrosis of rat nucleus pulposus cells under compression. Oxid Med Cell Longev.

[189] Luo R, Liao Z, Song Y, Yin H, Zhan S, Li G (2019). Berberine ameliorates oxidative stress-induced apoptosis by modulating ER stress and autophagy in human nucleus pulposus cells. Life Sci.

[190] Chen L, Liu L, Xie ZY, Wang F, Zhu L, Zhang C (2019). Protein kinase RNA-like ER kinase/eukaryotic translation initiation factor 2α pathway attenuates tumor necrosis factor alpha-induced apoptosis in nucleus pulposus cells by activating autophagy. J Cell Physiol.

[191] Chang H, Cai F, Zhang Y, Xue M, Liu L, Yang A (2017). Early-stage autophagy protects nucleus pulposus cells from glucose deprivation-induced degeneration via the p-eIF2α/ATF4 pathway. Biomed Pharmacother.

[192] Chino H, Mizushima N (2020). ER-phagy: quality control and turnover of endoplasmic reticulum. Trends Cell Biol.

[193] Luo R, Liang H, Zhang W, Li G, Zhao K, Hua W (2022). RETREG1-mediated ER-phagy activation induced by glucose deprivation alleviates nucleus pulposus cell damage via ER stress pathway. Acta Biochim Biophys Sin (Shanghai).

[194] Krupkova O, Sadowska A, Kameda T, Hitzl W, Hausmann ON, Klasen J (2018). p38 MAPK facilitates crosstalk between endoplasmic reticulum stress and IL-6 release in the intervertebral disc. Front Immunol.

[195] Wen T, Xue P, Ying J, Cheng S, Liu Y, Ruan D (2021). The role of unfolded protein response in human intervertebral disc degeneration: Perk and IRE1-α as two potential therapeutic targets. Oxid Med Cell Longev.

[196] Zhang J, Wang X, Liu H, Li Z, Chen F, Wang H (2019). TNF-α enhances apoptosis by promoting chop expression in nucleus pulposus cells: role of the MAPK and NF-κB pathways. J Orthop Res.

[197] Chen L, Liu L, Xie ZY, Wang F, Sinkemani A, Zhang C (2018). Endoplasmic reticulum stress facilitates the survival and proliferation of nucleus pulposus cells in TNF-α stimulus by activating unfolded protein response. DNA Cell Biol.

[198] Qadir A, Liang S, Wu Z, Chen Z, Hu L, Qian A (2020). Senile osteoporosis: the involvement of differentiation and senescence of bone marrow stromal cells. Int J Mol Sci.

[199] Yan J, Li S, Zhang Y, Deng Z, Wu J, Huang Z (2021). Cholesterol induces pyroptosis and matrix degradation via mSREBP1-driven endoplasmic reticulum stress in intervertebral disc degeneration. Front Cell Dev Biol.

[200] Hu MH, Yang KC, Chen YJ, Sun YH, Yang SH (2014). Lovastatin prevents discography-associated degeneration and maintains the functional morphology of intervertebral discs. Spine J.

[201] Tu J, Li W, Zhang Y, Wu X, Song Y, Kang L (2017). Simvastatin inhibits IL-1β-induced apoptosis and extracellular matrix degradation by suppressing the NF-κB and MAPK pathways in nucleus pulposus cells. Inflammation.

[202] Yu P, Zhang X, Liu N, Tang L, Peng C, Chen X (2021). Pyroptosis: mechanisms and Diseases. Signal Transduct Target Ther.

[203] Alvarez-Garcia O, Matsuzaki T, Olmer M, Miyata K, Mokuda S, Sakai D (2018). FOXO are required for intervertebral disk homeostasis during aging and their deficiency promotes disk degeneration. Aging Cell.

[204] Song Y, Wang Y, Zhang Y, Geng W, Liu W, Gao Y (2017). Advanced glycation end products regulate anabolic and catabolic activities via NLRP3-inflammasome activation in human nucleus pulposus cells. J Cell Mol Med.

[205] Song Y, Li S, Geng W, Luo R, Liu W, Tu J (2018). Sirtuin 3-dependent mitochondrial redox homeostasis protects against AGEs-induced intervertebral disc degeneration. Redox Biol.

[206] Luo R, Song Y, Liao Z, Yin H, Zhan S, Wang K (2019). Impaired calcium homeostasis via advanced glycation end products promotes apoptosis through endoplasmic reticulum stress in human nucleus pulposus cells and exacerbates intervertebral disc degeneration in rats. FEBS J.

[207] Urban JP, Smith S, Fairbank JC (2004). Nutrition of the intervertebral disc. Spine (Phila Pa 1976).

[208] Wang F, Cai F, Shi R, Wei JN, Wu XT (2016). Hypoxia regulates sumoylation pathways in intervertebral disc cells: implications for hypoxic adaptations. Osteoarthr Cartil.

[209] Zhu L, Xie ZY, Jiang ZL, Wang XH, Shi H, Chen L (2022). Unfolded protein response alleviates acid-induced premature senescence by promoting autophagy in nucleus pulposus cells. Cell Biol Int.

[210] Xie ZY, Chen L, Zhang C, Liu L, Wang F, Cai F (2018). Acid-sensing ion channel 1a regulates fate of rat nucleus pulposus cells in acid stimulus through endoplasmic reticulum stress. Biores Open Access.

[211] Iu J, Santerre JP, Kandel RA (2018). Towards engineering distinct multi-lamellated outer and inner annulus fibrosus tissues. J Orthop Res.

[212] Chu G, Shi C, Lin J, Wang S, Wang H, Liu T (2018). Biomechanics in annulus fibrosus degeneration and regeneration. Adv Exp Med Biol.

[213] Zhang YH, Zhao CQ, Jiang LS, Dai LY (2011). Cyclic stretch-induced apoptosis in rat annulus fibrosus cells is mediated in part by endoplasmic reticulum stress through nitric oxide production. Eur Spine J.

[214] Chen J, Lin Z, Deng K, Shao B, Yang D (2019). Tension induces intervertebral disc degeneration via endoplasmic reticulum stress-mediated autophagy. Biosci Rep.

[215] Pang L, Yang K, Zhang Z (2020). High-glucose environment accelerates annulus fibrosus cell apoptosis by regulating endoplasmic reticulum stress. Biosci Rep.

[216] Zhu Q, Gao X, Levene HB, Brown MD, Gu W (2016). Influences of nutrition supply and pathways on the degenerative patterns in human intervertebral disc. Spine (Phila Pa 1976).

[217] Han Y, Li X, Yan M, Yang M, Wang S, Pan J (2019). Oxidative damage induces apoptosis and promotes calcification in disc cartilage endplate cell through ROS/MAPK/NF-κB pathway: implications for disc degeneration. Biochem Biophys Res Commun.

[218] Deldicque L (2013). Endoplasmic reticulum stress in human skeletal muscle: any contribution to Sarcopenia?. Front Physiol.

[219] Anker SD, Morley JE, von Haehling S (2016). Welcome to the ICD-10 code for Sarcopenia. J Cachexia Sarcopenia Muscle.

[220] Cruz-Jentoft AJ, Baeyens JP, Bauer JM, Boirie Y, Cederholm T, Landi F (2010). Sarcopenia: European consensus on definition and diagnosis: report of the European working group on Sarcopenia in older people. Age Ageing.

[221] Cruz-Jentoft AJ, Bahat G, Bauer J, Boirie Y, Bruyere O, Cederholm T (2019). Sarcopenia: revised European consensus on definition and diagnosis. Age Ageing.

[222] Antunes AC, Araújo DA, Veríssimo MT, Amaral TF (2017). Sarcopenia and hospitalisation costs in older adults: a cross-sectional study. Nutr Diet.

[223] Meng SJ, Yu LJ (2010). Oxidative stress, molecular inflammation and sarcopenia. Int J Mol Sci.

[224] Narici MV, Maffulli N (2010). Sarcopenia: characteristics, mechanisms and functional significance. Br Med Bull.

[225] Jheng JR, Chen YS, Ao UI, Chan DC, Huang JW, Hung KY (2018). The double-edged sword of endoplasmic reticulum stress in uremic sarcopenia through myogenesis perturbation. J Cachexia Sarcopenia Muscle.

[226] Deldicque L, Hespel P, Francaux M (2012). Endoplasmic reticulum stress in skeletal muscle: origin and metabolic consequences. Exerc Sport Sci Rev.

[227] Barreiro E, Salazar-Degracia A, Sancho-Munoz A, Gea J (2019). Endoplasmic reticulum stress and unfolded protein response profile in quadriceps of sarcopenic patients with Respiratory Diseases. J Cell Physiol.

[228] Naidoo N (2009). ER and aging-protein folding and the ER stress response. Ageing Res Rev.

[229] Ogata T, Machida S, Oishi Y, Higuchi M, Muraoka I (2009). Differential cell death regulation between adult-unloaded and aged rat soleus muscle. Mech Ageing Dev.

[230] Pierre N, Barbé C, Gilson H, Deldicque L, Raymackers JM, Francaux M (2014). Activation of ER stress by hydrogen peroxide in C2C12 myotubes. Biochem Biophys Res Commun.

[231] Potes Y, de Luxan-Delgado B, Rodriguez-Gonzalez S, Guimaraes MRM, Solano JJ, Fernandez-Fernandez M (2017). Overweight in elderly people induces impaired autophagy in skeletal muscle. Free Radic Biol Med.

[232] Bodine SC, Latres E, Baumhueter S, Lai VK, Nunez L, Clarke BA (2001). Identification of ubiquitin ligases required for skeletal muscle atrophy. Science.

[233] Hwee DT, Baehr LM, Philp A, Baar K, Bodine SC (2014). Maintenance of muscle mass and load-induced growth in muscle RING finger 1 null mice with age. Aging Cell.

[234] Furuhashi M, Hotamisligil GS (2008). Fatty acid-binding proteins: role in metabolic Diseases and potential as drug targets. Nat Rev Drug Discov.

[235] Lee SM, Lee SH, Jung Y, Lee Y, Yoon JH, Choi JY (2020). FABP3-mediated membrane lipid saturation alters fluidity and induces ER stress in skeletal muscle with aging. Nat Commun.

[236] Tezze C, Romanello V, Desbats MA, Fadini GP, Albiero M, Favaro G (2017). Age-associated loss of OPA1 in muscle impacts muscle mass, metabolic homeostasis, systemic inflammation, and epithelial senescence. Cell Metab.

[237] Rennie MJ (2009). Anabolic resistance: the effects of aging, sexual dimorphism, and immobilization on human muscle protein turnover. Appl Physiol Nutr Metab.

[238] Rennie MJ, Selby A, Atherton P, Smith K, Kumar V, Glover EL (2010). Facts, noise and wishful thinking: muscle protein turnover in aging and human disuse atrophy. Scand J Med Sci Sports.

[239] Breen L, Phillips SM (2013). Interactions between exercise and nutrition to prevent muscle waste during ageing. Br J Clin Pharmacol.

[240] Deldicque L, Bertrand L, Patton A, Francaux M, Baar K (2011). ER stress induces anabolic resistance in muscle cells through PKB-induced blockade of mTORC1. PLoS ONE.

[241] Clark BC, Manini TM (2008). Sarcopenia =/= dynapenia. J Gerontol A Biol Sci Med Sci.

[242] Russ DW, Grandy JS, Toma K, Ward CW (2011). Ageing, but not yet senescent, rats exhibit reduced muscle quality and sarcoplasmic reticulum function. Acta Physiol (Oxf).

[243] Russ DW, Krause J, Wills A, Arreguin R (2012). SR stress in mixed hindlimb muscles of aging male rats. Biogerontology.

[244] Atkins JL, Whincup PH, Morris RW, Lennon LT, Papacosta O, Wannamethee SG (2014). Sarcopenic obesity and risk of Cardiovascular Disease and mortality: a population-based cohort study of older men. J Am Geriatr Soc.

[245] Bryner RW, Woodworth-Hobbs ME, Williamson DL, Alway SE (2012). Docosahexaenoic acid protects muscle cells from palmitate-induced atrophy. ISRN Obes.

[246] Woodworth-Hobbs ME, Perry BD, Rahnert JA, Hudson MB, Zheng B, Russ Price S (2017). Docosahexaenoic acid counteracts palmitate-induced endoplasmic reticulum stress in C2C12 myotubes: impact on muscle atrophy. Physiol Rep.

[247] Deldicque L, Cani PD, Philp A, Raymackers JM, Meakin PJ, Ashford ML (2010). The unfolded protein response is activated in skeletal muscle by high-fat feeding: potential role in the downregulation of protein synthesis. Am J Physiol Endocrinol Metab.

[248] Tang YH, Yue ZS, Zheng WJ, Shen HF, Zeng LR, Hu ZQ (2018). 4-phenylbutyric acid presents therapeutic effect on osteoarthritis via inhibiting cell apoptosis and inflammatory response induced by endoplasmic reticulum stress. Biotechnol Appl Biochem.

[249] Liu C, Cao Y, Yang X, Shan P, Liu H (2015). Tauroursodeoxycholic acid suppresses endoplasmic reticulum stress in the chondrocytes of patients with osteoarthritis. Int J Mol Med.

[250] Wang W, Qing X, Wang B, Ma K, Wei Y, Shao Z (2018). Tauroursodeoxycholic acid protects nucleus pulposus cells from compression-induced apoptosis and necroptosis via inhibiting endoplasmic reticulum stress. Evid Based Complement Alternat Med.

[251] Hamamura K, Lin CC, Yokota H (2013). Salubrinal reduces expression and activity of MMP13 in chondrocytes. Osteoarthr Cartil.

[252] Hamamura K, Tanjung N, Yokota H (2013). Suppression of osteoclastogenesis through phosphorylation of eukaryotic translation initiation factor 2 alpha. J Bone Miner Metab.

[253] Mullan LA, Mularczyk EJ, Kung LH, Forouhan M, Wragg JM, Goodacre R (2017). Increased intracellular proteolysis reduces Disease severity in an ER stress-associated dwarfism. J Clin Invest.

[254] Chen SZ, Ling Y, Yu LX, Song YT, Chen XF, Cao QQ (2021). 4-phenylbutyric acid promotes hepatocellular carcinoma via initiating cancer stem cells through activation of PPAR-α. Clin Transl Med.

[255] Gohlke H, Schmitz B, Sommerfeld A, Reinehr R, Häussinger D (2013). α5 β1-integrins are sensors for tauroursodeoxycholic acid in hepatocytes. Hepatology.

[256] Hamamura K, Nishimura A, Chen A, Takigawa S, Sudo A, Yokota H (2015). Salubrinal acts as a Dusp2 inhibitor and suppresses inflammation in anti-collagen antibody-induced arthritis. Cell Signal.

